# Meta-analysis of exome array data identifies six novel genetic loci for lung function

**DOI:** 10.12688/wellcomeopenres.12583.3

**Published:** 2018-08-07

**Authors:** Victoria E. Jackson, Jeanne C. Latourelle, Louise V. Wain, Albert V. Smith, Megan L. Grove, Traci M. Bartz, Ma'en Obeidat, Michael A. Province, Wei Gao, Beenish Qaiser, David J. Porteous, Patricia A. Cassano, Tarunveer S. Ahluwalia, Niels Grarup, Jin Li, Elisabeth Altmaier, Jonathan Marten, Sarah E. Harris, Ani Manichaikul, Tess D. Pottinger, Ruifang Li-Gao, Allan Lind-Thomsen, Anubha Mahajan, Lies Lahousse, Medea Imboden, Alexander Teumer, Bram Prins, Leo-Pekka Lyytikäinen, Gudny Eiriksdottir, Nora Franceschini, Colleen M. Sitlani, Jennifer A. Brody, Yohan Bossé, Wim Timens, Aldi Kraja, Anu Loukola, Wenbo Tang, Yongmei Liu, Jette Bork-Jensen, Johanne M. Justesen, Allan Linneberg, Leslie A. Lange, Rajesh Rawal, Stefan Karrasch, Jennifer E. Huffman, Blair H. Smith, Gail Davies, Kristin M. Burkart, Josyf C. Mychaleckyj, Tobias N. Bonten, Stefan Enroth, Lars Lind, Guy G. Brusselle, Ashish Kumar, Beate Stubbe, Understanding Society Scientific Group, Mika Kähönen, Annah B. Wyss, Bruce M. Psaty, Susan R. Heckbert, Ke Hao, Taina Rantanen, Stephen B. Kritchevsky, Kurt Lohman, Tea Skaaby, Charlotta Pisinger, Torben Hansen, Holger Schulz, Ozren Polasek, Archie Campbell, John M. Starr, Stephen S. Rich, Dennis O. Mook-Kanamori, Åsa Johansson, Erik Ingelsson, André G. Uitterlinden, Stefan Weiss, Olli T. Raitakari, Vilmundur Gudnason, Kari E. North, Sina A. Gharib, Don D. Sin, Kent D. Taylor, George T. O'Connor, Jaakko Kaprio, Tamara B. Harris, Oluf Pederson, Henrik Vestergaard, James G. Wilson, Konstantin Strauch, Caroline Hayward, Shona Kerr, Ian J. Deary, R. Graham Barr, Renée de Mutsert, Ulf Gyllensten, Andrew P. Morris, M. Arfan Ikram, Nicole Probst-Hensch, Sven Gläser, Eleftheria Zeggini, Terho Lehtimäki, David P. Strachan, Josée Dupuis, Alanna C. Morrison, Ian P. Hall, Martin D. Tobin, Stephanie J. London

**Affiliations:** 1 Department of Health Sciences, University of Leicester, Leicester, UK; 2 Department of Neurology, Boston University School of Medicine, Boston, MA, USA; 3 National Institute for Health Research, Leicester Respiratory Biomedical Research Unit, Glenfield Hospital, Leicester, UK; 4 Icelandic Heart Association, 201 Kopavogur, Iceland; 5 University of Iceland, 101 Reykjavik, Iceland; 6 Human Genetics Center, Department of Epidemiology, Human Genetics, and Environmental Sciences, School of Public Health, The University of Texas Health Science Center at Houston, Houston, TX, 77030, USA; 7 Cardiovascular Health Research Unit, Departments of Medicine and Biostatistics, University of Washington, Seattle, WA, 98101, USA; 8 The University of British Columbia Centre for Heart Lung Innovation, St Paul’s Hospital, Vancouver, BC, Canada; 9 Department of Genetics, Washington University School of Medicine, St. Louis, MO, USA; 10 Department of Biostatistics, Boston University School of Public Health, Boston, MA, USA; 11 Institute for Molecular Medicine Finland (FIMM), University of Helsinki, FI-00014, Helsinki, Finland; 12 Centre for Genomic & Experimental Medicine, MRC Institute of Genetics & Molecular Medicine, University of Edinburgh, Edinburgh, EH4 2XU, UK; 13 Division of Nutritional Sciences, Cornell University, Ithaca, NY, USA; 14 Department of Healthcare Policy and Research, Division of Biostatistics and Epidemiology, Weill Cornell Medical College, New York City, NY, USA; 15 Novo Nordisk Foundation Center for Basic Metabolic Research, Faculty of Health and Medical Sciences, University of Copenhagen, 2200 Copenhagen, Denmark; 16 Steno Diabetes Center Copenhagen, Gentofte, 2820, Denmark; 17 Department of Medicine, Division of Cardiovascular Medicine, Stanford University School of Medicine, Palo Alto, CA, USA; 18 Research Unit of Molecular Epidemiology, Institute of Epidemiology II, Helmholtz Zentrum München, German Research Center for Environmental Health, 85764 Neuherberg, Germany; 19 Medical Research Council Human Genetics Unit, Institute of Genetics and Molecular Medicine, University of Edinburgh, Edinburgh , EH4 2XU, UK; 20 Centre for Cognitive Ageing and Cognitive Epidemiology, University of Edinburgh, Edinburgh, EH8 9JZ, UK; 21 Centre for Genomic and Experimental Medicine, University of Edinburgh, Edinburgh, EH4 2XU, UK; 22 Center for Public Health Genomics, University of Virginia, Charlottesville, VA, USA; 23 Department of Medicine, College of Physicians and Surgeons, Columbia University, New York, NY, USA; 24 Department of Preventive Medicine - Division of Health and Biomedical Informatics, Northwestern University - Feinberg School of Medicine, Chicago, IL, USA; 25 Department of Clinical Epidemiology, Leiden University Medical Center, Leiden, 2333 ZA, Netherlands; 26 Department of Immunology, Genetics, and Pathology, Biomedical Center, SciLifeLab Uppsala, Uppsala University, SE-75108 Uppsala, Sweden; 27 Wellcome Trust Centre for Human Genetics, University of Oxford, Oxford, UK; 28 Respiratory Medicine, Ghent University Hospital, Ghent, BE9000, Belgium; 29 Bioanalysis, Ghent University, Ghent, BE9000, Belgium; 30 Swiss Tropical and Public Health Institute, Basel, Switzerland; 31 University of Basel, Basel, Switzerland; 32 Institute for Community Medicine, University Medicine Greifswald, 17475 Greifswald, Germany; 33 Human Genetics, Wellcome Trust Sanger Institute, Hinxton, CB10 1SA, UK; 34 Department of Clinical Chemistry, Fimlab Laboratories, Tampere 33520, Finland; 35 Department of Clinical Chemistry, Faculty of Medicine and Life Sciences, University of Tampere, Tampere 33014, Finland; 36 Department of Epidemiology, Gillings School of Global Public Health, University of North Carolina at Chapel Hill, NC 27514, USA; 37 Cardiovascular Health Research Unit, Department of Medicine, University of Washington, Seattle, WA, 98101, USA; 38 Institut universitaire de cardiologie et de pneumologie de Québec, Department of Molecular Medicine, Laval University, Québec, Canada; 39 Department of Pathology and Medical Biology, University Medical Center Groningen, University of Groningen, NL9713 GZ, Netherlands; 40 Groningen Research Institute for Asthma and COPD, University Medical Center Groningen, University of Groningen, Groningen, Netherlands; 41 Boehringer Ingelheim , Danbury, CT, USA; 42 Wake Forest School of Medicine, Winston-Salem, North Carolina, USA; 43 Centre for Clinical Research and Prevention, Bispebjerg and Frederiksberg Hospital, The Capital Region, Copenhagen, Denmark; 44 Department of Clinical Experimental Research, Rigshospitalet, 2600 Glostrup, Denmark; 45 Department of Clinical Medicine, Faculty of Health and Medical Sciences, University of Copenhagen, 2200 Copenhagen, Denmark; 46 Department of Medicine, Division of Bioinformatics and Personalized Medicine, University of Colorado Denver, Aurora, CO, USA; 47 Institute of Epidemiology I, Helmholtz Zentrum München, German Research Center for Environmental Health, 85764 Neuherberg, Germany; 48 Institute and Outpatient Clinic for Occupational, Social and Environmental Medicine, Ludwig-Maximilians-Universität, Munich, Germany; 49 Division of Population Health Sciences, Ninewells Hospital and Medical School, University of Dundee, Dundee, DD1 9SY, UK; 50 Department of Psychology, University of Edinburgh, Edinburgh, EH8 9JZ, UK; 51 Department of Pulmonology, Leiden University Medical Center, Leiden, 2333 ZA, Netherlands; 52 Department of Public Health and Primary Care, Leiden University Medical Center, Leiden, 2333 ZA, Netherlands; 53 Department of Medical Sciences, Uppsala University Hospital, Uppsala, Sweden; 54 Epidemiology, Erasmus Medical Center, Rotterdam, 3000CA, Netherlands; 55 Respiratory Medicine, Erasmus Medical Center, Rotterdam, 3000CA, Netherlands; 56 Institute of Environmental Medicine, Karolinska Institutet, Stockholm, Sweden; 57 Internal Medicine B, University Medicine Greifswald, Greifswald, 17475, Germany; 58 Department of Clinical Physiology, Tampere University Hospital, Tampere, 33521, Finland; 59 Department of Clinical Physiology, Faculty of Medicine and Life Sciences, University of Tampere, Tampere, 33014, Finland; 60 Epidemiology Branch, National Institute of Environmental Health Sciences, National Institutes of Health, Dept of Health and Human Services, Research Triangle Park, NC, 27709, USA; 61 Cardiovascular Health Research Unit, Departments of Epidemiology, Medicine and Health Services, University of Washington, Seattle, WA, 98101, USA; 62 Kaiser Permanente Washington Health Research Institute, Seattle, WA, USA; 63 Cardiovascular Health Research Unit, Department of Epidemiology, University of Washington, Seattle, WA, 98101, USA; 64 Department of Genetics and Genomic Sciences, Icahn School of Medicine at Mount Sinai, New York, NY, 10029-6574, USA; 65 Icahn Institute of Genomics and Multiscale Biology, Icahn School of Medicine at Mount Sinai, New York, NY, 10029-6574, USA; 66 Department of Health Sciences, University of Jyväskylä, Jyväskylä, Fl-40014, Finland; 67 Sticht Center on Aging, Wake Forest School of Medicine, Winston-Salem, NC, USA; 68 Comprehensive Pneumology Center Munich (CPC-M), Member of the German Center for Lung Research, Munich, Germany; 69 Faculty of Medicine, University of Split, Split, Croatia; 70 Alzheimer Scotland Research Centre, University of Edinburgh, Edinburgh, EH8 9JZ, UK; 71 Department of Medical Sciences, Molecular Epidemiology and Science for Life Laboratory, Uppsala University, Uppsala, Sweden; 72 Department of Medicine, Division of Cardiovascular Medicine, Stanford University School of Medicine, Stanford, CA, 94305, USA; 73 Internal Medicine, Erasmus Medical Center, Rotterdam, 3000CA, Netherlands; 74 Interfaculty Institute for Genetics and Functional Genomics, University Medicine Greifswald, Greifswald, 17475, Germany; 75 DZHK (German Centre for Cardiovascular Research), partner site: Greifswald, Greifswald, Germany; 76 Department of Clinical Physiology and Nuclear Medicine, Turku University Hospital, Turku, 20521, Finland; 77 Research Centre of Applied and Preventative Cardiovascular Medicine, University of Turku, Turku, 20014, Finland; 78 Department of Epidemiology and Carolina Center for Genome Science, University of North Carolina, Chapel Hill, NC, 27514, USA; 79 Computational Medicine Core, Center for Lung Biology, UW Medicine Sleep Center, Department of Medicine, University of Washington, Seattle, WA, 98109, USA; 80 Respiratory Division, Department of Medicine, University of British Columbia, Vancouver, BC, Canada; 81 Institute for Translational Genomics and Population Sciences and Department of Pediatrics, Los Angeles Biomedical Research Institute at Harbor-UCLA Medical Center, Torrance, CA, 90502, USA; 82 Pulmonary Center, Department of Medicine, Boston University School of Medicine, Boston, MA, 02118, USA; 83 National Heart, Lung and Blood Institute's and Boston University's Framingham Heart Study, Framingham, MA, 01702, USA; 84 Department of Health, University of Helsinki, Helsinki, FI-00014, Finland; 85 Department of Public Health, National Institute for Health and Welfare, Helsinki, FI-00271, Finland; 86 National Institute on Aging, National Institutes of Health, Bethesda, MD, 20892, USA; 87 Department of Physiology and Biophysics, University of Mississippi Medical Center, Jackson, MS, 39216, USA; 88 Institute of Genetic Epidemiology, Helmholtz Zentrum München, German Research Center for Environmental Health, Neuherberg, 85764, Germany; 89 Chair of Genetic Epidemiology, IBE, Faculty of Medicine, LMU Munich, Munich, 81377, Germany; 90 Department of Epidemiology, Mailman School of Public Health, Columbia University, New York, NY, 10032, USA; 91 Department of Biostatistics, University of Liverpool, Liverpool, L69 3GL, UK; 92 Radiology, Erasmus Medical Center, Rotterdam, 3000CA, Netherlands; 93 Neurology, Erasmus Medical Center, Rotterdam, 3000CA, Netherlands; 94 Department of Internal Medicine - Pulmonary Diseases, Vivantes Klinikum Spandau Berlin, Berlin, 13585, Germany; 95 Population Health Research Institute, St George's, University of London, London, SW17 0RE, UK; 96 NIHR Nottingham Biomedical Research Centre and Division of Respiratory Medicine, University of Nottingham, Nottingham, NG7 2UH, UK

**Keywords:** Lung function, respiratory, exome array, GWAS, COPD

## Abstract

**Background:** Over 90 regions of the genome have been associated with lung function to date, many of which have also been implicated in chronic obstructive pulmonary disease.

**Methods:** We carried out meta-analyses of exome array data and three lung function measures: forced expiratory volume in one second (FEV _1_), forced vital capacity (FVC) and the ratio of FEV _1_ to FVC (FEV _1_/FVC). These analyses by the SpiroMeta and CHARGE consortia included 60,749 individuals of European ancestry from 23 studies, and 7,721 individuals of African Ancestry from 5 studies in the discovery stage, with follow-up in up to 111,556 independent individuals.

**Results:** We identified significant (P<2·8x10 ^-7^) associations with six SNPs: a nonsynonymous variant in *RPAP1*, which is predicted to be damaging, three intronic SNPs ( *SEC24C, CASC17 *and *UQCC1*) and two intergenic SNPs near to *LY86 *and *FGF10.* Expression quantitative trait loci analyses found evidence for regulation of gene expression at three signals and implicated several genes, including *TYRO3* and *PLAU*.

**Conclusions: **Further interrogation of these loci could provide greater understanding of the determinants of lung function and pulmonary disease.

## Introduction

Measures of lung function act as predictors of mortality and morbidity and form the basis for the diagnosis of several diseases, most notably chronic obstructive pulmonary disease (COPD), one of the leading causes of death globally ^1^. Environmental factors, including smoking and exposure to air pollution play a significant role in lung function; however there has also been shown to be a genetic component, with estimates of the narrow sense heritability ranging between 39–66% ^2– 5^. Genome-wide association studies (GWAS) of lung function have identified associations between single nucleotide polymorphisms (SNPs) and lung function at over 150 independent loci to date ^6– 14^. Associations have also been identified in GWAS of COPD ^15– 19^; however, the identification of disease associated SNPs has been restricted by limited sample sizes. Many signals first identified in powerful studies of quantitative lung function traits, have been found to be associated with risk of COPD, highlighting the potential clinical usefulness of comprehensive identification of lung function associated SNPs ^13^.

Low frequency (minor allele frequency (MAF) 1–5%) and rare (MAF<1%) variants have been largely underexplored by GWAS to date. Exome arrays have been designed to facilitate the investigation of these low frequency and rare variants, predominately within coding regions, in large sample sizes. Alongside a core content of rare coding SNPs, the exome array additionally includes common variation, including tags for previously identified GWAS hits, ancestry informative SNPs, a grid of markers for estimating identity by descent and a random selection of synonymous SNPs ^20^.

An earlier version of this article can be found on bioRxiv ( https://doi.org/10.1101/164426)

## Results

We carried out a meta-analysis of exome array data and three lung function measures: forced expiratory volume in one second (FEV _1_), forced vital capacity (FVC) and the ratio of FEV _1_ to FVC (FEV _1_/FVC). These analyses included 68,470 individuals from the SpiroMeta and CHARGE consortia in a discovery analysis, with follow-up in an independent sample of up to 111,556 individuals. All studies are listed with their study-specific sample characteristics in Table 1, with full study descriptions, including details of spirometry and other measurements described in the Supplementary Note. The genotype calling procedures implemented by each study ( Supplementary Table 1) and quality control of genotype data are described in the Supplementary Methods. We have undertaken both single variant analyses, and gene-based associations, which test for the joint effect of several rare variants in a gene (see *Methods* for details).

**Table 1.** Sample characteristics of 11 SpiroMeta and 12 CHARGE studies contributing to the discovery analyses and three studies contributing to the replication analyses.

Discovery studies							
SpiroMeta studies	Total sample	n (%) Male	Ever smokers, n (%)	Age, mean (SD)	FEV _1_, litres. mean (SD)	FVC, litres. mean (SD)	FEV _1_/FVC, mean (SD)
1958 British Birth Cohort (B58C)	5270	2961 (56·2%)	2866 (53·3%)	44·00 (0·00)	3·35 (0·79)	4·29 (1·03)	0·788 (0·09)
Generation Scotland (GS:SFHS)	8164	3413 (41·8%)	3806 (46·6%)	51·59 (13·33)	2·78 (0·87)	3·91 (1·01)	0·710 (0·12)
Cooperative Health Research in the Region of Augsburg (KORA F4)	1447	701 (48·5%)	900 (62·2%)	54·82 (9·66)	3·24 (0·85)	4·20 (1·04)	0·771 (0·07)
CROATIA-Korcula cohort (KORCULA)	791	296 (36·8%)	418 (52·0%)	55·56 (13·69)	2·72 (0·83)	3·29 (0·95)	0·829 (0·10)
Lothian Birth Cohort 1936 (LBC1936)	974	501 (50·6%)	554 (55·9%)	69·55 (0·84)	2·38 (0·67)	3·04 (0·87)	0·787 (0·10)
Study of Health in Pomerania (SHIP)	1681	831 (49·4%)	955 (56·8%)	52·25 (13·43)	3·29 (0·88)	3·88 (1·03)	0·848 (0·07)
Northern Swedish Population Health Study (NSPHS)	880	407 (46·3%)	122 (13·9%)	49·13 (19·96)	2·93 (0·90)	3·53 (1·06)	0·831 (0·09)
Prospective Investigation of the Vasculature in Uppsala Seniors (PIVUS)	836	413 (49·4%)	426 (51·0%)	70·20 (0·17)	2·44 (0·68)	3·20 (0·87)	0·76 (0·10)
Swiss study on Air Pollution and Lung Disease in adults (SAPALDIA)	2707	1379 (50·9%)	1399 (51·7%)	40·86 (10·92)	3·65 (0·83)	4·62 (1·04)	0·794 (0·07)
The Cardiovascular Risk in Young Finns Study (YFS)	434	198 (47·3%)	186 (44·4%)	38·88 (5·07)	3·73 (0·75)	4·68 (0·99)	0·800 (0·06)
Finnish Twin Cohort (FTC)	214	0 (0%)	0 (0%)	68·73 (3·31)	2·18 (0·47)	2·79 (0·58)	0·786 (0·08)
**Total**	**23,398**						
CHARGE studies (European Ancestry)	Total sample	n (%) Male	Ever smokers, n (%)	Age, mean (SD)	FEV _1_, litres. mean (SD)	FVC, litres. mean (SD)	FEV _1_/FVC, mean (SD)
AGES-Reykjavik study (AGES)	1566	649 (41·4%)	900 (57·5%)	76·1 (5·62)	2·13 (0·70)	2·87 (0·86)	0·744 (0·09)
Atherosclerosis Risk in Communities Study (ARIC)	10,680	5015 (47·0%)	631 (59·1%)	54·3 (5·70)	2·94 (0·77)	3·98 (0·98)	0·738 (0·07)
Cardiovascular Health Study (CHS)	3967	1737 (43·8%)	2089 (52·7%)	72·8 (5·55)	2·11 (0·66)	3·00 (0·86)	0·702 (0·10)
NHLBI Family Heart Study (FAMHS)	1651	718 (43·5%)	698 (42·3)	53·5 (12·60)	2·91 (0·853)	3·89 (1·05)	0·746 (0·08)
Framingham Heart Study (FHS)	7113	3241 (45·5%)	3780 (53·1)	50·7 (14·12)	3·10 (0·925)	4·09 (1·12)	0·755 (0·08)
Health Aging and Body Composition Study (HABC)	1457	786 (53·2%)	831 (56·5%)	73·7 (2·83)	2·31 (0·66)	3·11 (0·81)	0·741 (0·08)
Health2006 Study	2714	1217 (44·8%)	1577 (58·1%)	49·4 (13·04)	3·13 (0·82)	3·99 (0·99)	0·784 (0·07)
Health2008 Study	687	297 (43·2%)	384 (55·9%)	46·7 (8·22)	3·27 (0·79)	4·13 (0·97)	0·791 (0·06)
Inter99 Study (without pack-years)	1115	549 (49·2%)	1115 (100%)	47·2 (7·76)	3·26 (0·71)	4·12 (0·92)	0·796 (0·07)
Inter99 Study (with pack-years)	4179	2027 (48·5%)	2307 (55·2%)	45·8 (7·95)	3·21 (0·76)	4·10 (0·97)	0·788 (0·08)
Multi-Ethnic Study of Atherosclerosis (MESA)	1323	654 (49·4%)	751 (56·8%)	66·0 (9·8)	2·57 (0·76)	3·51 (0·10)	0·733 (0·08)
The Rotterdam Study (RS)	546	299 (54·8%)	382 (70·0%)	79·4 (5·00)	2·27 (0·68)	3·03 (0·86)	0·750 (0·08)
**Total**	**36,998**						
CHARGE studies (African Ancestry)	Total Sample	n (%) Male	Ever smokers, n (%)	Age, mean (SD)	FEV _1_, litres. mean (SD)	FVC, litres. mean (SD)	FEV _1_/FVC, mean (SD)
Atherosclerosis Risk in Communities Study (ARIC)	3180	1183 (37·2%)	1680 (59·1%)	53·6 (5·83)	2·48 (0·65)	3·25 (0·82)	0·765 (0·08)
Cardiovascular Health Study (CHS)	624	232 (37·2%)	340 (54·4%)	73·2 (5·49)	1·76 (0·58)	2·48 (0·80)	0·717 (0·11)
Health Aging and Body Composition Study (HABC)	943	433 (45·9%)	543 (57·6%)	73·4 (2·90)	1·96 (0·57)	2·61 (0·71)	0·749 (0·09)
Jackson Heart Study (JHS)	2143	793 (36·8%)	688 (31·9%)	52·8 (12·6)	2·43 (0·72)	3·02 (0·86)	0·807 (0·09)
Multi-Ethnic Study of Atherosclerosis (MESA)	861	404 (46·9%)	467 (54·2%)	65·6 (9·6)	2·19 (0·66)	2·92 (0·86)	0·756 (0·09)
**Total**	**7721**						
Replication studies							
Study name	Total Sample	n (%) Male	Ever smokers, n (%)	Age, mean (SD)	FEV _1_, litres. mean (SD)	FVC, litres. mean (SD)	FEV _1_/FVC, mean (SD)
UK Biobank	98,657	45,166 (45·8%)	56,404 (57·2%)	56·7 (7·92)	2·75 (0·80)	3·67 (0·98)	0·75 (0·07)
UK Household Longitudinal Study (UKHLS)	7443	3293 (44·2%)	4509 (60·5%)	53·10 (15·94)	2·89 (0·90)	3·83 (1·08)	0·753 (0·09)
Netherlands Epidemiology of Obesity study (NEO)	5456	2672 (48·0%)	3674 (66·0%)	55·9 (5·9)	3·26 (0·80)	4·26 (1·02)	0·77 (0·07)
**Total**	**111,556**						

### Meta-analyses of single variant associations

We first evaluated single variant associations between FEV _1_, FVC and FEV _1_/FVC and the 179,215 SNPs that passed study level quality control and were polymorphic in both consortia. These analyses identified 34 SNPs in regions not previously associated with lung function, showing association with at least one trait at overall P<10 ^-5^, and showing association with consistent direction and P<0·05 in both consortia (full results in Supplementary Table 2, quantile-quantile and Manhattan plots shown in Supplementary Figure 1). We followed up these SNP associations in a replication analysis comprising 3 studies with 111,556 individuals. Combining the results from the discovery and replication stages in a meta-analysis identified six SNPs in total that were independent to known signals and met the pre-defined significance threshold (P<2·8×10 ^-7^) overall in, or near to *FGF10*, *LY86*, *SEC24C*, *RPAP1, CASC17* and *UQCC1* ( Table 2, Supplementary Figure 2). A SNP near to the *CASC17* signal (rs11654749, r ^2^=0·3 with rs1859962) has previously been associated with FEV _1_ in a genome-wide analysis of gene-smoking interactions, although this association was not replicated at the time ^21^; the present analysis provides the first evidence for independent replication of this signal. A seventh signal was also identified in *LCT* ( Table 2, Supplementary Figure 2); whilst this locus has not previously been implicated in lung function, this SNP is known to vary in frequency across European populations ^22^, and we cannot rule out that this association is not an artefact of population structure. Our discovery analysis furthermore identified associations (P<10 ^-5^) in 25 regions previously associated with one or more of FEV _1_, FVC and FEV _1_/FVC ( Supplementary Table 3).

**Table 2.** Novel loci associated with lung function traits. Results are shown for variant in novel loci associated (P<2·7×10 ^-7^) with lung function traits in a two stage meta-analysis consisting of up to 68,470 individuals from the SpiroMeta and CHARGE Consortia in the discovery analyses, with follow-up in up to 111,556 individuals from UK Biobank, UKHLS and NEO. For each SNP, the result for the trait-smoking-ancestry combination which resulted in the most statistically significant association is given. The results for these SNPs and all three traits are shown in Supplementary Table 12. Beta values from SpiroMeta (β _Sp_) reflect effect-size estimates on an inverse-normal transformed scale after adjustments for age, age ^2^, sex, height and ancestry principal components, and stratified by ever smoking status (Analysis of All individuals only). Beta values from CHARGE (β _CH_) reflect effect-size estimates on an untransformed scale (litres for FEV _1_ and FVC; ratio for FEV _1_/FVC). Samples sizes (N), Z-statistics (Z) and two-sided P-values (P) are given for the combined discovery analysis and the replication analysis. Two-sided P-values are also given for the full two-stage combined analyses (discovery + replication).

								Consortium results		Combined discovery meta-analysis			Replication			Two-stage combined
SNP	Chr:Pos	(Nearest) gene(s)	Trait	Smoking	Ancestry	Effect/other allele	Effect allele frequency (Discovery)	β _CH_	β _Sp_	N _disc_	Z _disc_	P _disc_	N _rep_	Z _rep_	P _rep_	P _meta_
rs2322659	2:136555659	LCT (nonsynonymous)	FVC	All Individuals	EA Only	T/C	23·5%	27·34	0·032	55,591	5·597	2·18×10 ^-8^	12,899	2·286	0·0223	1·70 ×10 ^-9^
rs1448044	5:44296986	*FGF10*(dist=8111), *NNT*(dist=591,318)	FVC	Ever Smokers	EA+AA	A/G	35·6%	18·63	0·057	30,966	4·813	1·49 ×10 ^-6^	64,400	4·805	1·55 ×10 ^-6^	2·22 ×10 ^-11^
rs1294421	6:6743149	*LY86*(dist=87,933), *RREB1*(dist=364,681)	FEV _1_/ FVC	All Individuals	EA+AA	T/G	36·8%	-0·222	-0·038	68,099	-5·479	4·27 ×10 ^-8^	111,556	-8·171	3·06 ×10 ^-16^	9·74 ×10 ^-23^
rs3849969	10: 75525999	*SEC24C* (intronic)	FEV1	All Individuals	EA+AA	T/C	29·4%	13·10	0·036	68,116	4·767	1·87 ×10 ^-6^	111,556	5·042	4·60 ×10 ^-7^	4·99 ×10 ^-12^
rs1200345	15: 41819716	*RPAP1* (nonsynonymous)	FEV1/ FVC	All Individuals	EA only	C/T	48·8%	-0·217	-0·025	60,381	-4·586	4·51 ×10 ^-6^	111,556	-5·725	1·03 ×10 ^-8^	2·33 ×10 ^-13^
rs1859962	17: 69108753	*CASC17* (intronic)	FEV _1_	All Individuals	EA only	G/T	48·2%	15·39	0·026	60,395	4·876	1·08 ×10 ^-6^	111,554	4·612	3·99 ×10 ^-6^	4·10 ×10 ^-11^
rs6088813	20: 33975181	*UQCC1* (intronic)	FVC	All Individuals	EA+AA	C/A	36·7%	-16·16	-0·023	68,115	-4·634	3·58×10 ^-6^	111,556	-7·688	1·50 ×10 ^-14^	4·90 ×10 ^-19^

Generally, the observed effect of the SNPs at the novel signals were similar in ever and never smokers; the exception was rs1448044 near *FGF10*, which showed a significant association with FVC only in ever smokers in our discovery analysis (ever smokers P=1·49×10 ^-6^; never smokers P=0·695, Supplementary Table 4 and Supplementary Figure 3). In the replication analysis, however, this association was observed in both ever and never smokers (ever smokers P=3·14×10 ^-5^; never smokers P=1·40×10 ^-4^, Supplementary Table 5). For rs1200345 ( *RPAP1*) and rs1859962 ( *CASC17*), associations were most statistically significant in the analyses restricted to individuals of European Ancestry ( Supplementary Table 4 and Supplementary Figure 3), as was the association with rs2322659 ( *LCT*), giving further support that this association may be due to population stratification.

### Meta-analyses of gene-based associations

We undertook Weighted Sum Tests (WST) ^23^ and Sequence Kernel Association tests (SKAT) ^24^ to assess the joint effects of multiple low frequency variants within genes on lung function traits. In our discovery analyses of all 68,470 individuals, we tested up to 14,380 genes that had at least two variants with MAF<5% and met the inclusion criteria (exonic or loss of function [LOF], see *Methods* for definitions) in both consortia. The SKAT analyses identified 16 genes associated (P<0·05 in both consortia and overall P<10 ^-4^) with FEV _1_, FVC or FEV _1_/FVC ( Supplementary Table 6), whilst the WST analyses identified 12 genes ( Supplementary Table 7). There was one gene ( *LY6G6D*) that was identified in both analyses. These genes were followed up in UK Biobank, with two genes, *GPR126* and *LTBP4,* showing evidence of replication in the exonic SKAT analysis (P<3·5×10 ^-6^); however conditional analyses in UK Biobank showed that both these associations were driven by single SNPs, that were identified in the single variant association analyses and have been previously reported in GWAS of these traits ( Supplementary Table 6 and Supplementary Table 7).

### Functional characterization of novel loci

In order to gain further insight into the six loci identified in our analyses of single variant associations (excluding *LCT*), we employed functional annotation and assessed whether identified SNPs in these regions were associated with gene expression levels. One of the identified novel SNPs was nonsynonymous, three intronic and two were intergenic. We found evidence that three of the SNPs may be involved in cis-acting regulation of the expression of several genes in multiple tissues ( Supplementary Table 8).

SNP rs1200345 in *RPAP1* is a nonysynomous variant, predicted to be deleterious by both SIFT (deleterious) and Polyphen (possibly damaging) ( Supplementary Table 9); *RPAP1* is ubiquitously expressed, with ** high levels of protein detected in the lung ( Supplementary Table 10). SNP rs1200345 or proxies (r ^2^>0·8) were also found to be amongst the most strongly associated SNPs with expression levels of *RPAP1* in several tissues, including lung, and with a further six genes in lung tissue ( Supplementary Table 8), including *TYRO3,* one of the TAM family of receptor tyrosine kinases. *TYRO3* regulates several processes including cell survival, migration and differentiation and is highly expressed in lung macrophages ( Supplementary Table 10). Evidence of association with gene expression was found at two more of the novel signals (sentinel SNPs rs3849969 and rs6088813), implicating a further 16 genes. Of note, in blood expression quantitative trait loci (eQTL) databases, a proxy of a SNP in complete linkage disequilibrium (r ^2^=1) with rs3849969 (rs3812637) was an eQTL for plasminogen activator, urokinase ( *PLAU*).

## Discussion

We undertook an analysis of 68,470 individuals from 23 studies with data from the exome array and three lung function traits, following up the most significant single SNP and gene-based associations in an independent sample of up to 111,556 individuals. There were six SNPs which reached P<10 ^-5^ in the discovery stage meta-analysis of single variant associations, and subsequently met the Bonferroni corrected significance threshold for independent replication (P<1⋅47×10 ^-3^, corrected for 34 SNPs being tested). In the combined analyses of our discovery and replication analyses, these six SNPs met the exome chip-wide significance threshold (P<2⋅8×10 ^-7^). One of the SNPs is in a region that has previously been implicated in lung function (near *KCJN2/SOX9*) ^21^, whilst the remaining five SNPs, although all common, have not previously been identified in other GWAS of lung function. In a recent 1000 Genomes imputed analysis of lung function (which includes some of the studies contributing to the present discovery analysis), all of these SNPs showed at least suggestive association (2·97×10 ^-3^>P>1·28×10 ^-5^) with one or more lung function trait, but none reached the required threshold (P<5×10 ^-6^) to be taken forward for replication in that analysis ^12^.

We further identified a seventh association with rs2322659 in *LCT* (MAF=23⋅5%; combined discovery + replication P=1⋅70×10 ^-9^). Given SNPs in this region are known to vary in frequency across European populations, we cannot dismiss the possibility that this association may be confounded by population stratification; hence we do not report this signal as a novel lung function locus. For SNPs at 7 loci that have been shown to have differences in allele frequency between individuals from different regions of the UK ^25^, and subsequently European populations (including the *LCT* locus), we undertook a look-up of associations with lung function in our discovery analyses. and subsequently across European populations ^26^. Aside from the association between the *LCT* locus and FVC, no significant associations were observed between SNPs at these loci and any lung function trait, in either the analyses restricted to European Ancestry (EA) individuals, or in the analysis of EA and African Ancestry (AA) individuals combined ( Supplementary Table 11); this suggests population structure was generally accounted for adequately in our analyses.

One of the novel signals was with a nonsynonymous SNP, rs1200345 in *RPAP1*, (MAF=48⋅8%; P=2⋅33×10 ^-13^), which is predicted to be deleterious. This SNP and proxies with r ^2^>0·8 were also associated with expression in lung tissue of seven genes, including *RPAP1* and the TAM receptor *TYRO3*. TAM receptors play a role in the inhibition of Toll-like receptors (TLRs)-mediated innate immune response by initiating the transcription of cytokine signalling genes (SOCS-1 and 3), which limit cytokine overproduction and inflammation ^27, 28^. It has been shown that influenza viruses H5N1 and H7N9 can cause downregulation of Tyro3, resulting in an increased inflammatory cytokine response ^28^.

Three further signals were with intronic SNPs in *SEC24C* (MAF=29⋅4%; P=4⋅99×10 ^-12^), *CASC17* (MAF=48⋅2%; P=4⋅10×10 ^-11^), and *UQCC1* (MAF=36⋅7%; P=4⋅90×10 ^-19^). Two of these intronic SNPs have previously been implicated in GWAS of other traits: rs1859962 in *CASC17* with prostate cancer ^29^ and rs6088813 in *UQCC1* with height ^30^. The *CASC17* locus, near *KCNJ2/SOX9* has also previously been implicated in lung function, showing significant association with FEV _1_ in a genome-wide analysis of gene-smoking interactions; however, this association was not formally replicated ^21^. Whilst the individuals utilised in the discovery stage of this analysis overlap with those included in this previous interaction analysis, the replication stage of the present study provides the first evidence of replication for this signal in independent cohorts. In the present analysis, there was no evidence that the results differed by smoking status.

SNPs rs6088813 in *UQCC1* and rs3849969 in *SEC24C* were identified as eQTLs for multiple genes. Whilst our eQTL analysis did not include formal tests of colocalisation, a SNP in complete linkage disequilibrium with rs3849969 (rs3812637, r ^2^=1) was associated with expression of *PLAU* in blood. The plasminogen activator, urokinase (PLAU) plays a role in fibrinolysis and immunity, and with its receptor (PLAUR) is involved in degradation of the extra cellular matrix, cell migration, cell adhesion and cell proliferation ^31^. A study of preterm infants with respiratory distress syndrome, a condition characterised by intra-alveolar fibrin deposition, found *PLAU* and its inhibitor *SERPINE1* to be expressed in the alveolar epithelium, and an increased ratio of SERPINE1 to PLAU was associated with severity of disease ^32^. Studies in mice have also shown that increased expression of *Plau* may be protective against lung injury, by reducing fibrosis ^33^. *PLAU* has also been found to be upregulated in lung epithelial cells subjected to cyclic strain ^34^ and in patients with COPD and lung cancer, PLAU was found to be expressed in alveolar macrophages and epithelial cells ^31^.

The final two signals were with common intergenic SNPs close to *LY86* (MAF=36⋅8%; P=9⋅74×10 ^-23^) and *FGF10* (MAF=35⋅6%; P=2⋅22×10 ^-11^). *LY86* (lymphocyte antigen 86) ** interacts with the Toll-like receptor signalling pathway, to form a heterodimer, when bound with RP105 ^35^. The sentinel SNP in the present analysis (rs1294421) has previously shown association with waist-hip ratio ^36^, whilst an intronic SNP within *LY86* (rs7440529, r ^2^=0·005 with rs1294421) has been implicated in asthma in two studies of individuals of Han Chinese ancestry ^37, 38^. FGF10 is a member of the fibroblast growth factor family of proteins, and is involved in a range of biological processes, including embryonic development and morphogenesis, cell growth and repair, tumor growth and invasion. Specifically, the FGF10 signalling pathway is thought to play an criticial role in the development of the lung and in lung epithelial renewal ^39^. A deficiency in *Fgf10* has been demonstrated to lead to a fatal disruption of branching morphogenesis during lung development in mice ^40^.

Our discovery analyses included individuals of both EA and AA. Two of the identified six novel signals showed inconsistent effects in the AA and EA individuals. For these SNPs, the associations in AA individuals were not statistically significant, and we report associations from the analysis restricted to EA individuals only. For the remaining four SNPs similar effects were observed in both the EA and AA individuals ( Supplementary Figure 3). We also examined the effects of the novel SNPs in ever smokers and never smokers separately and found these to be broadly similar, with the exception of rs1448044 in *FGF10,* which in the discovery analysis showed significant association with FVC in ever smokers, whilst showing no association in never smokers (P=0·695). However, in our replication stage analyses, similar effects were seen in both ever and never smokers for this SNP, and the combined analysis of discovery and replication stages for this SNP, including both ever and never smokers, met the exome chip-wide significance level overall (P=4·22×10 ^-9^). We also considered whether this signal could be driven by smoking behaviour in our discovery stage as our primary analyses in SpiroMeta did not adjust for smoking quantity. We undertook a look-up of this SNP in the publicly available results of a GWAS of several smoking behaviour traits ^41^; there was only weak evidence that this SNP was associated with ever versus never smoking (P=0·039), and no evidence for association with amount smoked (cigarettes per day, P=0·10).

Through the use of the exome array, we aimed to identify associations with low frequency and rare functional variants, thereby potentially uncovering some of the missing heritability of lung function. However, whilst our discovery analyses identified single SNP associations with 23 low frequency variants ( Supplementary Table 2), we did not replicate any of these findings. Eleven of these 23 SNPs we were unable to follow-up in our replication studies, due to them either being not genotyped, or monomorphic. Overall, limited statistical power is likely to explain our lack of convincing single variant associations with rare variants, in particular if those variants exhibit only modest effects ^42^. We additionally investigated the joint effects of low frequency and rare variants within genes, on lung function trait, by employing SKAT and WST gene-based tests. These analyses identified associations with a number of genes that could not attributed to the effect of a single SNP. Replication of these gene-based signals proved difficult however, as again a number of SNPs included in the discovery stage of these analyses were monomorphic, or had not been not genotyped in the replication studies. This lead to a disparity in the gene unit being tested in our discovery and replication samples; hence interpretation of these results was not clear-cut. In the end, we were able to replicate only findings with common SNPs. This finding is in line with several other studies of complex traits and exome array data that have been unable to report robust associations with low frequency variants ^43– 45^ and it is clear that future studies will require increasingly larger sample sizes in order to fully evaluate the effect of variants across the allele frequency spectrum. The identification of common SNPs remains important, however, as such findings have the potential to highlight drug targets ^46^, and these variants collectively could have utility in risk prediction.

In our replication analyses using UK Biobank, we applied adjustment for covariates including ancestry principal components, before undertaking inverse-normal transformations of the lung function phenotypes. Association analyses were then performed using these transformed phenotypes. It has recently been shown that such transformation has the potential to introduce correlations between principal components and phenotypes ^47^; we undertook sensitivity analyses for the six reported SNPs by repeating the association analyses with phenotypes that had been transformed without prior adjustment, with covariate adjustment made as part of the SNP-trait association test. We found there to be some difference in P-values for some SNP-trait combinations; however, the six novel SNP associations we report all met the replication P-value threshold (P<1·47×10 ^-3^) in the sensitivity analyses ( Supplementary Figure 4). This issue may also be relevant to the gene-based tests; however no replicated novel gene-based associations were identified in this study. Future studies should avoid undertaking adjustment for principal components of ancestry prior to trait transformation, in order to avoid this potential bias.

This study has identified six common SNPs, independent to signals previously implicated in lung function. Additional interrogation of these loci could lead to greater understanding of lung function and lung disease, and could provide novel targets for therapeutic interventions.

## Methods

### Study design, cohorts and genotyping

**Figure 1.**
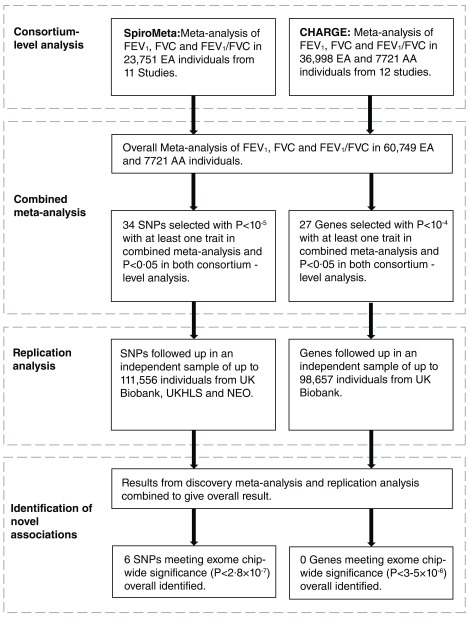
Study design.

The SpiroMeta analysis included 23,751 individuals of EA from 11 studies, and the CHARGE analysis comprised 36,998 EA individuals and a further 7,721 individuals of AA from 12 studies. Follow-up analyses were conducted in an independent sample of up to 111,556 individuals from UK Biobank (2015 interim release), the UK Household Longitudinal Study (UKHLS) and the Netherlands Epidemiology of Obesity (NEO) Study ( Figure 1). All studies (excluding UK Biobank) were genotyped using either the Illumina Human Exome BeadChip v1 or the Illumina Infinium HumanCoreExome-12 v1·0 BeadChip. UK Biobank samples were genotyped using the Affymetrix Axiom UK BiLEVE or UK Biobank arrays.

### Statistical analyses

**Consortium level analyses:** Within the SpiroMeta Consortium, each study contributing to the discovery analyses calculated single-variant score statistics, along with covariance matrices describing correlations between variants, using RAREMETALWORKER ^48^ or rvtests ^49^. For each trait, these summary statistics were generated separately in ever and never smokers. Traits were adjusted for sex, age, age ^2^ and height, and inverse normally transformed prior to association testing. For studies with unrelated individuals, SNP-trait associations were tested using linear models, with adjustments made for the first 10 ancestry principal components, whilst studies with related individuals utilised linear mixed models to account for familial relationships and underlying population structure.

Within the CHARGE Consortium, each study generated equivalent summary statistics using the R package SeqMeta ^50^. For each trait, summary statistics were generated in ever and never smokers separately, and in all individuals combined. The untransformed traits were used for all analyses, adjusted for smoking status and pack-years, age, age ^2^, sex, height, height ^2^, centre/cohort. Models for FVC were additionally adjusted for weight. Linear regression models, with adjustment for principal components of ancestry were used for studies with unrelated individuals, and linear mixed models were used for family-based studies.

Within each consortium we used the score statistics and variance-covariance matrices generated by each study to construct both single variant and gene-based tests using either RAREMETAL ^48^ (SpiroMeta) or SeqMeta ^50^ (CHARGE). For single variant associations, score statistics were combined in fixed effects meta-analyses. Two gene-based tests were constructed: first, the Weighted Sum Test (WST) using Madsen Browning weightings ^23^, and secondly, the Sequence Kernel Association Test (SKAT) ^24^. We performed the SKAT and WST tests using two subsets of SNPs: 1) including all SNPs with an overall consortium-wide MAF<5% that were annotated as splicing, stopgain, stoploss, or frameshift (loss of function [LOF] analysis), and 2) including all SNPs meeting the LOF analysis criteria in addition to all other nonsynonymous variants with consortium wide MAF<5% (exonic analysis). Variants were annotated to genes using dbNSFP v2·6 ^51^ on the basis of the GRCh37/hg19 database.

For both single variant and gene-based associations, consortium-level results were generated for ever smokers and never smokers separately, and in all individuals combined. Within the CHARGE Consortium, results were combined separately for the EA and AA studies and also in a trans-ethnic analysis of both ancestries.

**Combined meta-analysis:** The single variant association results from the SpiroMeta and CHARGE consortia were combined as follows: The genomic inflation statistic (λ) was calculated for SNPs with consortium-wide MAF>1%; where λ had a value greater than one, genomic control adjustment was applied to the consortium level P-values. The consortium-level results were then combined using sample size weighted z-score meta-analysis. The λ was again calculated for the meta-analysis results and genomic control applied, as appropriate. λ values at the consortium and meta-analysis level are shown in Supplementary Table 13. Since we were interested in identifying low frequency and rare variants, we applied no MAF or minor allele count (MAC) filter. We identified SNPs of interest as those with an overall P<10 ^-5^ and a consistent direction of effect and P<0·05 observed in both consortia. Rather than using a strict Bonferroni correction for defining the significance threshold, we adopted the more lenient P<10 ^-5^ threshold in order to increase the power to detect variants with modest effect in our discovery analyses, whilst the requirement for consistency in results from the two consortia aimed to limit false positives. All SNPs meeting these thresholds were followed up in independent replication cohorts. Where we identified a SNP within 1Mb of a previously identified lung function SNP, we deemed the SNP to represent an independent signal if it had r ^2^<0·2 with the known SNP, and if it retained a P <10 ^-5^, when conditional analyses were carried out with the known SNP, or a genotyped proxy, using data from the SpiroMeta Consortium, or UK Biobank. Our primary meta-analysis included all individuals; we additionally carried out analyses in smoking subgroups (ever and never smokers), and in the subgroup of individuals of European ancestry only.

For genes which contained at least 2 polymorphic SNPs in both consortia, we combined the results of the consortium level gene based tests using either z-score meta-analysis (for the WST analysis) or Fisher’s Method for combining P-values (in the case of SKAT). We identified genes of interest as those with P<0·05 observed in both consortia and an overall P<10 ^-4^, thresholds again chosen to limit both false positive and false negative findings. As in the analyses of single variant associations, our primary meta-analyses included all individuals, with secondary analyses undertaken in smoking and ancestry specific subgroups.

**Replication analyses:** All SNP and gene-based associations were followed up for the trait with which they showed the most statistically significant association only. For associations identified through the smoking subgroup analyses, we followed up associations in the appropriate smoking strata; however, no ancestry stratified follow-up was undertaken as replication studies included only a sufficient number of individuals of European Ancestry.

Single variant associations in UK Biobank were tested in ever smokers and never smokers separately, and stratified by genotyping array (UK BiLEVE array or UK Biobank array) using the score test as implemented in SNPTEST v2·5b4 ^52^. Traits were adjusted for age, age ^2^, height, sex, ten principal components and pack-years (ever smokers only), and the adjusted traits were inverse normally transformed. Correlations between principal components and transformed phenotypes may be introduced where adjustment is made prior to transformation. In this analysis, we found any introduced correlations to have no impact on the conclusion of our replication analyses; however future studies should apply transformation of phenotypes prior to covariate adjustment, to avoid this issue. For UKHLS, analyses were undertaken analogously to the SpiroMeta discovery studies using RAREMETALWORKER, while for NEO, analyses were undertaken in the same way as was done in the CHARGE discovery studies using SeqMeta. The single variant results from all replication studies were combined using sample size weighted Z-score meta-analysis. Subsequently, we combined the results from the discovery and replication stage analyses and we report SNPs with overall exome-wide significance of P<2·8×10 ^-7^ (Bonferroni corrected for the original 179,215 SNPs tested).

We followed up genes of interest (P<10 ^-4^) using data from UK Biobank only. Summary statistics for UK Biobank were generated using RAREMETALWORKER, with gene-based tests then constructed using RAREMETAL. Finally, we combined the results from the discovery analysis with the replication results in an overall combined meta-analysis using either z-score meta-analysis (WST) or Fisher’s Method (SKAT). We declared genes with overall P<3·5×10 ^-6^ (Bonferroni corrected for 14,380 genes tested) in our combined meta-analysis to be statistically significant. For these statistically significant genes, we carried out additional analyses using the UK Biobank data in which we conditioned on the most significantly associated individual SNP within that gene, to determine whether this was a true gene-based signal, or whether the association could be ascribed to the single SNP (if the conditional P<0·01, then association was deemed to not be driven by the single SNP).

### Characterization of findings

In order to gain further insight into the loci identified in our analyses of single variant associations, we assessed whether these regions were associated with gene expression levels in various tissues (FDR of 5%, or q-value<0·05), by querying a publically available blood eQTL database ^53^ and the GTEx project ^54^ for the sentinel SNPs, or any proxy (r ^2^>0·8). We further assessed SNPs of interest (and proxies) within a lung eQTL resource based on non-tumour lung tissues of 1,111 individuals ^55– 57^. Descriptions of these resources and further details of the look-ups are provided in the Supplementary Methods. Moreover, all sentinel SNPs and proxies with r ^2^>0.8 were annotated using ENSEMBL’s Variant Effect Predictor (VEP) ^58^; potentially deleterious coding variants were identified as those annotated as ‘deleterious’ by SIFT ^59^ or ‘probably damaging’ or ‘possibly damaging’ by PolyPhen-2 ^60^. For all genes implicated through the expression data or functional annotation, we searched for evidence of protein expression in the respiratory system by querying the Human Protein Atlas ^61^.

## Data availability

Summary level results for all analyses are available on OSF: https://doi.org/10.17605/OSF.IO/NSDPJ ^62^

Data are available under the terms of the Creative Commons Attribution 4.0 International license (CC-BY 4.0).

This research has been conducted using the UK Biobank Resource. The genetic and phenotypic UK Biobank data are available upon application to the UK Biobank ( https://www.ukbiobank.ac.uk/) to all registered health researchers. These data are from Understanding Society: The UK Household Longitudinal Study (UKHLS), which is led by the Institute for Social and Economic Research at the University of Essex and funded by the Economic and Social Research Council. The data were collected by NatCen and the genome wide scan data were analysed by the Wellcome Trust Sanger Institute. Information on how to access the data can be found on the Understanding Society website https://www.understandingsociety.ac.uk/.

## Supplementary material

Supplementary Information: File includes Supplementary Note, Supplementary Methods, Supplementary Figures and Supplementary Tables, as detailed below.

**Supplementary Note** includes individual study descriptions.

**Supplementary Methods** includes details of study level quality control procedures and eQTL analyses.

**Supplementary Figures:**

Supplementary Figure 1 - Quantile-quantile (QQ) and Manhattan plots for consortium-wide analyses, and the combined meta-analysis.

Supplementary Figure 2 - Region Plots for novel loci.

Supplementary Figure 3 - Forest Plots for novel loci.

Supplementary Figure 4 - Trait Transformation Sensitivity Analysis.

**Supplementary Tables:**

Supplementary Table 1 - Details of study specific genotyping platform, genotype calling procedure and software.

Supplementary Table 2 - Association results for all SNPs identified in single variant association discovery analyses (P<10 ^-4^).

Supplementary Table 3 - Association results for SNPs identified in single variant association discovery analyses (P<10 ^-4^), located in known lung function regions.

Supplementary Table 4 - Single variant association result for the seven novel signals, in smoking and ancestry subgroups.

Supplementary Table 5 - Single variant association result for rs1448044 and FVC in ever smokers and never smokers separately, and in all samples combined.

Supplementary Table 6 - Association results for all genes identified in discovery SKAT analyses (meta-analysis P<10 ^-4^).

Supplementary Table 7 - Association results for all genes identified in discovery Weighted sum test (WST) test analyses (P<10 ^-4^).

Supplementary Table 8 - Evidence for the role of novel variants identified in single variant association analyses as eQTLs.

Supplementary Table 9 - SIFT/Polyphen predictions for sentinel SNPs and proxies (r2>0.8).

Supplementary Table 10 - Protein and RNA expression results all implicated genes from the single variant association analyses.

Supplementary Table 11 - Look-up of association results for SNPs at 7 of the 12 loci which showed allele frequency differences between individuals from different regions in the UK.

Supplementary Table 12 - All traits results for the seven novel lung function loci.

Supplementary Table 13 - Genomic Inflation Factors: consortium and meta-analysis level.
