# Supplementary material for: Meta-analysis of exome array data identifies six novel genetic loci for lung function
Source: Wellcome Open Res. 2018 Aug 7;3:4. Originally published 2018 Jan 12. [Version 3] doi: 10.12688/wellcomeopenres.12583.3 (PMC6081985; doi:10.12688/wellcomeopenres.12583.3)
Supplement: Supplementary file 1 [file wellcomeopenres-3-16020-s0000.tgz › 8cc3bea4-d873-4e51-9da3-a81b6b88afcb.pdf]

Supplementary Information: Meta-analysis of exome array data identifies six novel genetic loci for lung function

Victoria E Jackson, Jeanne C Lautourelle, Louise V Wain, Albert V. Smith, Megan L. Grove, Traci M Bartz, Maen Obeidat, Michael A Province, Wei Gao, Beenish Qaiser, David J Porteous, Patricia A Cassano, Tarunveer S. Ahluwalia, Niels Grarup, Jin Li, Elisabeth Altmaier, Jonathan Marten, Sarah E Harris, Ani Manichaikul, Tess D Pottinger, Ruifang Li-Gao, Allan Lind-Thomsen, Anubha Mahajan, Lies Lahousse, Medea Imboden, Alexander Teumer, Bram Prins, Leo-Pekka Lyytikäinen, Gudny Eiriksdottir, Nora Franceschini, Colleen M Sitlani, Jennifer A Brody, Yohan Bossé, Wim Timens, Aldi Kraja, Anu Loukola, Wenbo Tang, Yongmei Liu, Jette Bork-Jensen, Johanne M. Justesen, Allan Linneberg, Leslie A. Lange, Rajesh Rawal, Stefan Karrasch, Jennifer E Huffman, Blair H Smith, Gail Davies, Kristin M Burkart, Josyf C Mychaleckyj , Tobias N. Bonten, Stefan Enroth, Lars Lind, Guy G Brusselle, Ashish Kumar, Beate Stubbe, Understanding Society Scientific Group , Mika Kähönen, Annah B. Wyss, Bruce M Psaty, Susan R Heckbert, Ke Hao, Taina Rantanen, Stephen B Kritchevsky, Kurt Lohman, Tea Skaaby, Charlotta Pisinger, Torben Hansen, Holger Schulz, Ozren Polasek, Archie Campbell, John M Starr, Stephen S Rich, Dennis O. Mook-Kanamori, Åsa Johansson, Erik Ingelsson, André G Uitterlinden, Stefan Weiss, Olli T. Raitakari, Vilmundur Gudnason, Kari E. North, Sina A Gharib, Don D Sin, Kent D Taylor, George T O'Connor, Jaakko Kaprio, Tamara B Harris, Oluf Pedersen, Henrik Vestergaard, James G. Wilson, Konstantin Strauch, Caroline Hayward, Shona Kerr, Ian J Deary, R Graham Barr, Renée de Mutsert, Ulf Gyllensten, Andrew P Morris, M. Arfan Ikram, Nicole M Probst-Hensch, Sven Gläse, Eleftheria Zeggini, Terho Lehtimäki, David P Strachan, Josee Dupuis, Alanna C. Morrison, Ian P Hall, Martin D Tobin, Stephanie J London

Supplementary Note

Individual study descriptions

This section describes study-specific characteristics, and details of spirometric and other measurements. All participants provided written informed consent and studies were approved by local Research Ethics Committees and/or Institutional Review boards.

The Reykjavik Study cohort originally comprised a random sample of 30,795 men and women born in 1907–1935 and living in Reykjavik in 1967. A total of 19381 attended, resulting in 71% recruitment rate. The study sample was divided into six groups by birth year and birth date within month. One group was designated for longitudinal follow-up and was examined in all stages. One group was designated a control group and was not included in examinations until 1991. Other groups were invited to participate in specific stages of the study. Between 2002 and 2006, the **AGES-Reykjavik study (AGES)** re-examined 5764 survivors of the original cohort who had participated before in the Reykjavik Study.

Atherosclerosis Risk in Communities (ARIC), is a population based study of risk factors for atherosclerosis and its sequelae[1] in adults from four U.S. field centers aged 45-64 at recruitment in 1987-1989. ARIC spirometry measurements were made with a Collins Survey II water-seal spirometer (Collins Medical, Inc.) and Pulmo-Screen II software (PDS Healthcare Products, Inc.).

Details of the **British 1958 Birth Cohort (1958BC)** biomedical follow-up have been previously reported[2]. Spirometry at age 44–45 years was performed in the standing position without nose clips, using a Vitalograph handheld spirometer as previously described[3]. In the analysis, all readings with a best-test variation greater than 10% were excluded.

The **Cardiovascular Health Study (CHS)** is a population-based cohort study of risk factors for coronary heart disease and stroke in adults ≥65 years conducted across four field centers.[4] The original predominantly European ancestry cohort of 5,201 persons was recruited in 1989-1990 from random samples of the Medicare

eligibility lists; subsequently, an additional predominantly African-American cohort of 687 persons was enrolled in 1992-1993 for a total sample of 5,888. The baseline exam consisted of a clinic examination that included assessment of height and weight and self-reported smoking information. Pulmonary function testing was conducted by trained spirometry technicians. FEV_1/FVC and FEV_1 measures met American Thoracic Society criteria for acceptability. The pulmonary function measures analyzed were from the baseline visit for the original cohort and from one year after baseline for the second cohort. Measurements were made with a Collins Survey I water-seal spirometer (Collins Medical, Inc.) and software from S&M Instruments.[5] Blood samples were drawn from all participants at their baseline examination and DNA was subsequently extracted from available samples. Genotyping was performed at the General Clinical Research Center's Phenotyping/Genotyping Laboratory at Cedars-Sinai among CHS participants who consented to genetic testing and had DNA available using the Illumina HumanExome BeadChip v1.0 (the "Exome Chip") using standard protocols. Cryptically related individuals were identified and only one individual from each related group was retained for analysis.

The CROATIA study was initiated to investigate the use of isolated rather than urban populations for the identification of genes associated with medically-relevant quantitative traits. Three cohorts have been recruited as part of the CROATIA study, of which one, **CROATIA-Korcula** [6] has been used in these analyses. CROATIA-Korcula was recruited from 2007 to 2008 from the town of Korcula and the villages of Lumbarda, Zrnovo and Racisce on the island of Korcula, Croatia with 969 adults aged 18-98 agreeing to participate. Participants donated blood for DNA extraction and biochemical measurements as well as undergoing some anthropometric measurements and physiological tests to measure traits such as height, weight and blood pressure, and finally completing several questionnaires relating to general health, medical history, diet and lifestyle. Ethical approval was obtained from appropriate regulatory bodies in both Scotland and Croatia and participants gave informed consent prior to joining the study.

Details of the design of the **NHLBI Family Heart Study (FAMHS)** have been described previously. [7]Spirometry was performed during the participant's clinical examination using a computerized volume-based spirometer and was reported at body temperature and pressure, saturated. FEV_1 and FVC were measured.

The Framingham Heart Study (FHS) is a longitudinal community-based family study that originated in 1948 with the recruitment of adults from the town of Framingham, MA. The offspring of the original cohort were recruited to participate in 1971, and the third generation (Gen3) was recruited starting in 2002. Spirometry has been measured on all three generations of the participating families as part of the clinical examinations. For the original and offspring cohorts, spirometry was performed using a 6-L water-filled Collins survey spirometer connected to an Eagle II microprocessor (Collins Medical, Braintree, MA) or in later offspring examinations to a personal computer running software developed by S&M Instruments, Doylestown, PA. For the Gen3 cohort, spirometry was performed using the CPL System 53 (Collins Medical, Braintree, MA) with a dry rolling-seal spirometer. All of these systems provided an automatic correction for body temperature and pressure, saturated, and provided quality assurance information.

The **Finnish Twin Cohort (FTC)** sample originates from The Finnish Twin Study of Ageing (FITSA) sub-study. Participants were recruited from the older Finnish Twin Cohort for a clinical study of functional limitations in older women. Clinical assessment was conducted in 2000-2001 at the University of Jyväskylä. The final sample consisted of 103 monozygotic (MZ) and 114 dizygotic (DZ) twin pairs. Lung function was measured in the standing position using an electronic spirometer (Medikro 202, Kuopio, Finland). The subject was asked to inhale maximally and to exhale as fast as possible into a mouthpiece connected to a flow transducer and a flow-volume curve was created. At least two tests were performed and the best result taken for the analyses. Spirometer was calibrated daily with a three-litre pump and was accurate to within 1%.

The **Generation Scotland: Scottish Family Health Study** (**GS:SFHS**)is a collaboration between the Scottish Universities and the NHS, funded by the Chief Scientist Office of the Scottish Government. GS:SFHS is a family-

based genetic epidemiology cohort with DNA, other biological samples (serum, urine and cryopreserved whole blood) and socio-demographic and clinical data from ~24,000 volunteers, aged 18-98 years, in ~7,000 family groups. Participants were recruited across Scotland, with some family members from further afield, from 2006-2011. Most (87%) participants were born in Scotland and 96% in the UK or Ireland. The cohort profile has been published[8]. GS:SFHS operates under appropriate ethical approvals, and all participants gave written informed consent. Generation Scotland is a collaboration between the University Medical Schools and National Health Service in Aberdeen, Dundee, Edinburgh and Glasgow (UK).

The **Health Aging and Body Composition (HABC) study** is a prospective observational cohort of well-functioning individuals aged 70–79 years, which recruited 3,075 community-dwelling African and European Americans, men and women, at two field centers at the University of Pittsburgh, Pennsylvania and the University of Tennessee, Memphis. Spirometry was performed with a horizontal dry rolling seal spirometer (SensorMedics Corporation, Yorba Linda, CA) connected to a computer. Pulmonary function testing followed ATS guidelines for the standardization of spirometry, and is described in detail elsewhere.[9] Health ABC genotyped 1,794 self-described white participants and 1,281 self-described black participants at baseline with available DNA and consent to genetics testing; of these 1,661 white participants and 1,139 black participants passed quality control benchmarks (call rate > 97%, no sex mismatch, and cryptic relatedness), and 1,457 and 943, respectively, had pulmonary function measurements and complete data on covariates.

Health2006 is a population-based epidemiological study of general health, diabetes and cardiovascular disease of individuals aged 18-74 years conducted at the Research Centre for Prevention and Health in Glostrup, Denmark.[10] Spirometry was performed with the Spiro USB Spirometer (MicroMedical Limited, Rochester, Kent, UK). Measurements of forced expiratory volume in one second (FEV1) and forced vital capacity (FVC) were recorded.

Health2008 is an extension of the Health2006 study, where a random sample of the general population aged 30 to 60 years from the same regional areas in Copenhagen County was drawn from the Civil Registration System. Methodology was the same as in Health2006.

The **Inter99** study carried out in 1999-2001 included invitation of 12,934 persons aged 30-60 years drawn from an age- and sex-stratified random sample of the population.[11] The baseline participation rate was 52.5%, and the study included 6784 persons. The Inter99 study was a population-based randomized controlled trial (CT00289237, ClinicalTrials.gov) and investigated the effects of lifestyle intervention on CVD. Spirometry was performed with the Cardiosoft® software (GE Medical Systems, Freiburg, Germany) and an LF501 respiration flow transducer (Erich Jaeger B.V. and Marquette Hellige GmbH, Freiburg, Germany). Each morning, the spirometers were checked with a 3-L syringe. Measurements of forced expiratory volume in one second (FEV_1) and forced vital capacity (FVC) were recorded. Spirometry was performed by trained healthcare professionals and repeated until the participant was assumed to comply satisfactorily with the equipment. In the current analysis, the cohort was split into individuals with information on number of smoking pack years (**Inter99p**) and individuals without this information (**Inter99np**).

The **Jackson Heart Study (JHS)**[12] is a prospective population-based study to seek the causes of the high prevalence of common complex diseases among African Americans in the Jackson, Mississippi metropolitan area. During the baseline examination period (2000-2004) 5,301 self-identified African Americans were recruited from four sources, including (1) randomly sampled households from a commercial listing; (2) ARIC participants; (3) a structured volunteer sample that was designed to mirror the eligible population; and (4) a nested family cohort. Spirometry was performed as recommended by the American Thoracic Society[13] with a dry rolling seal spirometer (Occupational Marketing, Houston, Tex). Measurements included forced vital capacity (FVC), forced expiratory volume at 1 second (FEV1), 3 seconds, and 6 seconds, and peak flow as well as flows at 25%, 50%, and 75% of total volume. Both calculated data and raw wave-form data were stored for each forced exhalation performed, and quality scores for the test were developed and attached to each

participant's data. Unrelated participants were between 35 and 84 years old, and members of the family cohort were ≥ 21 years old when consent for genetic testing was obtained and blood was drawn for DNA extraction. A total of 2,790 JHS participants were genotyped on the Illumina Human Exome BeadChip v1. Participants that are also in the ARIC study were excluded for the current analysis.

The KORA studies (Cooperative Health Research in the Region of Augsburg) are a series of independent population based studies from the general population living in the region of Augsburg, Southern Germany[14,15]. **KORA F4** including 3,080 individuals was conducted from 2006-2008 as a follow-up study to KORA S4 (1999-2001). Lung function tests were performed in a random subsample of subjects born between 1946 and 1965 (age range 41–63 years). Spirometry was performed in line with the ATS/ERS recommendations[16] using a pneumotachograph-type spirometer (Masterscreen PC, CardinalHealth, Würzburg, Germany) before and after inhalation of 200µg salbutamol. The present study is based on maximum values of FEV_1 and FVC measured before bronchodilation. The spirometer was calibrated daily using a calibration pump (CardinalHealth, Würzburg, Germany), and additionally, an internal control was used to ensure constant instrumental conditions. For KORA F4 participants without spirometry measurements in 2006-2008 (n=126), we used measurements from the KORA-Age time point conducted in 2008/09. KORA Age contains subjects from all KORA studies born until 1943 (aged 65-90 years)[17]. Spirometry was measured in 935 randomly selected participants. Conditions including the examiner were the same as in 2008/09 except that inhalation of salbutamol was not performed due to the high number of contraindications anticipated in this aged population.

The **Lothian Birth Cohort 1936 (LBC1936)** consists of 1,091 relatively healthy individuals assessed on cognitive and medical traits at about 70 years of age. They were all born in 1936 and most took part in the Scottish Mental Survey of 1947. At baseline the sample of 548 men and 543 women had a mean age 69.6 years (s.d. = 0.8). They were all Caucasian, community-dwelling, and almost all lived in the Lothian region (Edinburgh city and surrounding area) of Scotland. A full description of participant recruitment and testing can be found elsewhere[18]. Genotyping was performed at the Wellcome Trust Clinical Research Facility, Edinburgh. Quality control measures were applied and 983 participants remained. Lung function assessing peak expiratory flow rate, forced expiratory volume in 1 second, and forced vital capacity (each the best of three), using a Micro Medical Spirometer was assessed, sitting down without nose clips, at age 70 years. The accuracy of the spirometer is ±3% (to ATS recommendations Standardisation of Spirometry 1994 update for flows and volumes).

The Multi-Ethnic Study of Atherosclerosis (**MESA**) is an NHLBI-sponsored population-based longitudinal investigation of subclinical cardiovascular disease and its progression.[19] In brief, a total of 6,814 individuals, aged 45 to 84 years at baseline, were recruited from six US communities between 2000 and August 2002. Thirty-eight percent of the originally recruited participants were White, 28% African-American, 22% Hispanic, and 12% Asian (predominantly of Chinese descent). Five in-person follow-up examinations have occurred since the MESA cohort was established. The MESA Air Pollution Study recruited an additional 257 participants.[20] Spirometry was conducted in 2004-06 in accordance with the American Thoracic Society/European Respiratory Society guidelines[21] on a dry-rolling-sealed spirometer with automated quality checks (Occupational Marketing, Inc., Houston, TX), as previously described,[22] for 3,975 participants. Pack-years of cigarette smoking were calculated as age of starting to quitting (or current age if current cigarette smoker) × (cigarettes per day/20) using standardized questionnaire items. Ever-smoking was defined as greater than 100 lifetime cigarettes smoked and current smoking as self-report of a cigarette in the last 30 days. Current smoking status was confirmed by cotinine levels in the subset of the cohort with spirometry measures; self-report was generally accurate.[23] Asthma was defined as self-report of physician-diagnosed asthma. Height was measured to the nearest 0.1 cm with the subject in stocking feet and weight was measured to the nearest pound with the subject in light clothing using a balanced scale.

The **Netherlands Epidemiology of Obesity (NEO) study** was designed for extensive phenotyping to investigate pathways that lead to obesity-related diseases. The NEO study is a population-based, prospective cohort study that includes 6,671 individuals aged 45–65 years, with an oversampling of individuals with overweight or obesity. At baseline, information on demography, lifestyle, and medical history have been collected by questionnaires. In addition, samples of 24-h urine, fasting and postprandial blood plasma and serum, and DNA were collected. Genotyping was performed using the Illumina HumanCoreExome chip, which was subsequently imputed to the 1000 genome reference panel. Participants underwent an extensive physical examination, including anthropometry, electrocardiography, spirometry, and measurement of the carotid artery intima-media thickness by ultrasonography. In random subsamples of participants, magnetic resonance imaging of abdominal fat, pulse wave velocity of the aorta, heart, and brain, magnetic resonance spectroscopy of the liver, indirect calorimetry, dual energy X-ray absorptiometry, or accelerometer measurements were performed. The collection of data started in September 2008 and completed at the end of September 2012. Participants are currently being followed for the incidence of obesity-related diseases and mortality.

The **Northern Sweden Population Health Study** (**NSPHS**) represents a cross-sectional study conducted in the communities of Karesuando (samples gathered in 2006) and Soppero (2009) in the subarctic region of the County of Norrbotten, Sweden. Spirometry was performed in sitting position without noseclips using a MicroMedicalSpida 5 spirometer (http://www. medisave.co.uk). Three consecutive lung function measurements per participant were done and the maximum value per measured lung function parameter was used for further analysis.

The **Prospective Investigation of the Vasculature in Uppsala Seniors** (**PIVUS**)[24] is a population-based study of the cardiovascular health in the elderly. The main purpose of PIVUS was to investigate the role endothelial function in cardiovascular risk. Mailed invitations were sent to subjects who lived in Uppsala, Sweden, within 2 months after their 70th birthday. The subjects were randomly selected from the community register. A total of 1,016 men and women participated in the baseline investigation (participation rate, 50.1%). Spirometry was performed in 901 subjects at baseline in accordance with American Thoracic Society recommendations (α spirometer; Vitalograph Ltd; Buckingham, UK). The best value from three recordings was used. The Ethics Committee of the University of Uppsala approved the study, and the participants gave their informed consent.

The **Rotterdam Study (RS)** is an ongoing prospective population-based cohort study, focused on chronic disabling conditions of the elderly. The study comprises an outbred ethnically homogenous population of Dutch Caucasian origin. The rationale of the study has been described in detail elsewhere.[25] In summary, 7,983 men and women aged 55 years or older, living in Ommoord, a suburb of Rotterdam, the Netherlands, were invited to participate. Spirometry was performed from 2009 onwards using a Master Screen® PFT Pro (CareFusion, San Diego, CA) by trained paramedical personnel according to the ATS/ERS guidelines.[26] A total of 546 individuals from the initial study with validated lung function measurement and exome chip data were included in the current study.

The **SAPALDIA** cohort is a population-based multi-center study in eight geographic areas representing the range of environmental, meteorological and socio-demographic conditions in Switzerland.[27,28] It was initiated in 1991 (SAPALDIA 1) with a follow-up assessment in 2002 (SAPALDIA 2) and 2010 (SAPALDIA3). This study has specifically been designed to investigate longitudinally lung function, respiratory and cardiovascular health; to study and identify the associations of these health indicators with individual long term exposure to air pollution, other toxic inhalants, life style and molecular factors.

The **Study of Health in West Pomerania** (**SHIP**) is a cross-sectional, population based survey in a region in the Northeast of Germany. Study details are given elsewhere[29,30]. The examinations were conducted using a bodyplethysmograph equipped with a pneumotachograph (VIASYS Healthcare, JAEGER, Hoechberg, Germany) which meets the American Thoracic Society (ATS) criteria[31]. The volume signal of the equipment was calibrated with a 3.0 litre syringe connected to the pneumotachograph in accordance with the manufacturer´s

recommendations and at least once on each day´s testing. Barometric pressure, temperature and relative humidity were registered every morning. Calibration of reference gas and volume was examined under ATS-conditions (Ambient Temperature Pressure) and the integrated volumes were BTPS (Body Temperature Pressure Saturated) corrected[31,32]. Lung function variables were measured continuously throughout the baseline breathing and the forced manoeuvres using a VIASYS HEALTHCARE system (MasterScreen Body/Diff.). Spirometry flow volume loops were conducted in accordance with ATS recommendations in a sitting position and with wearing noseclips. The participants performed at least three forced expiratory lung function manoeuvres in order to obtain a minimum of two acceptable and reproducible values. Immediate on-screen error codes indicating the major acceptability (including start, duration and end of test) and reproducibility criteria supported the attempt for standardised procedures. The procedure was continuously monitored by a physician. The best results for FVC, FEV_1, peak expiratory flow (PEF) and expiratory flow at 75%, 50%, 25% of FVC (MEF 75, MEF 50, MEF 25) were taken. The ratio of FEV_1 to FVC was calculated from the largest FEV_1 and FVC.

The **UK Biobank** samples comprised of 48,930 individuals selected for the UK Biobank Lung Exome Variant Evaluation (UK BiLEVE) project[33] and a further 49,727 samples selected randomly from UK Biobank, whom had high quality spirometry data.
UK Biobank (http://www.ukbiobank.ac.uk/) contains data from 502,682 individuals (94% of self-reported European ancestry) with extensive health and lifestyle questionnaire data, physical measures (including spirometry) and DNA. Spirometry was undertaken using a Vitalograph Pneumotrac 6800. The participant was asked to record two to three blows (lasting for at least 6 seconds) within a period of about 6 minutes. The computer compared the reproducibility of the first two blows and, if acceptable (defined as a <5% difference in forced volume vital capacity (FVC) and Forced Expiratory Volume in 1 second (FEV_1), a third blow was not required. For the UK BiLEVE project, a sampling frame of 275,939 individuals was defined as those who were of European ancestry and had spirometry measures which met ERS/ATS guidelines [16]. A total of 50,008 samples were selected from the extremes and middle of the distributions of percent predicted FEV_1, separately in never smokers and heavy smokers (20,005 individuals with low FEV_1, 19,997 with average FEV_1 and 10,006 with high FEV_1). DNA was extracted and genotyped with the custom-designed Affymetrix Axiom UK BiLEVE array. A further 102,757 individuals from UK Biobank, chosen at random, have subsequently been genotyped using the Affymetrix Axiom UK Biobank array; from these we selected 51,117 individuals of European ancestry and with spirometry meeting ATS/ERS guidelines. All UK BiLEVE and UK Biobank individuals were imputed to the 1000 Genomes Project Phase 1[45] and UK10K[46,47] combined panel and following quality control of the imputed genotype data and removal of related individuals, a total of 98,657 individuals remained for association analysis.

The **United Kingdom Household Longitudinal Study (UKHLS)**, also known as Understanding Society (https://www.understandingsociety.ac.uk) is a longitudinal panel survey of 40,000 UK households (England, Scotland, Wales and Northern Ireland) representative of the UK population. Participants are surveyed annually since 2009 and contribute information relating to their socioeconomic circumstances, attitudes, and behaviours via a computer assisted interview. The study includes phenotypical data for a representative sample of participants for a wide range of social and economic indicators as well as a biological sample collection encompassing biometric, physiological, biochemical, and haematological measurements and self-reported medical history and medication use. The United Kingdom Household Longitudinal Study has been approved by the University of Essex Ethics Committee and informed consent was obtained from every participant.
For a subset of individuals who took part in a nurse health assessment, blood samples were taken and genomic DNA extracted. Of these, 10,484 samples were genotyped at the Wellcome Trust Sanger Institute using the Illumina Infinium HumanCoreExome-12 v1.0BeadChip.
Lung function measures in samples from England and Wales were conducted with the NDD Easy On-PC spirometer (NDD Medical Technologies, Zurich, Switzerland). Participants were excluded in the following cases:

pregnancy, having had abdominal or chest surgery (past 3 weeks), admitted to the hospital with a heart complaint (in the past 6 weeks), having had recent eye surgery (past 4 weeks), or in case of having a tracheostomy. Subjects were asked to perform up to 8 blows that ideally lasted at least 6 seconds, uninterrupted by coughing, glottis closure, laughing or leakage of air. Upon completion, the measurements were rated either acceptable or unacceptable by the NDD Easy On-PC software.

The **Young Finns Study** (**YFS)** is a population-based follow up-study started in 1980[34]. The main aim of the YFS is to determine the contribution made by childhood lifestyle, biological and psychological measures to the risk of cardiovascular diseases in adulthood. In 1980, over 3,500 children and adolescents all around Finland participated in the baseline study. The follow-up studies have been conducted mainly with 3-year intervals. The latest 30-year follow-up study was conducted in 2010-2011 (ages 33-49 years) with 2,063 participants. The study was approved by the local ethics committees (University Hospitals of Helsinki, Turku, Tampere, Kuopio and Oulu) and was conducted following the guidelines of the Declaration of Helsinki. All participants gave their written informed consent.

Supplementary Methods

Study level Quality Control Procedures

For B58C, KORA F4, NSPHS, PIVUS, SAPALDIA, SHIP, FIN and YFS, genotype calling and quality control were carried out in accordance with the Exome Chip Quality Control SOP Version 5, as developed within the UK exome chip consortium.[35] Genotypes were initially called using Gencall in Illumina's Genome Studio software.[36] Quality control of SNPs and samples was subsequently performed at study level. Initial filters applied excluded SNPs with very low call rate (<90%) and samples with low call rate, heterozygosity outliers, duplicates, gender mismatches and ancestral outliers. SNPs with missing data were then recalled using genotype calling software zCall (zCall not implemented in YFS).[37] All alleles were mapped to the forward strand of human genome build 37 and secondary exclusions were applied to remove SNPs with low call rate (<99%) or deviations from Hardy Weinberg Equilibrium ($P<10^{-4}$). Samples with call rate <99% and heterozygosity outliers were also excluded.

For AGES; ARIC; CHS; FAMHS; FHS; HABC; Health2006; Health 2008; Inter99np; Inter99p; JHS; MESA; RS, GS:SFHS, KORCULA and LBC1936, genotypes were called using Gencall in Illumina's Genome Studio software[36] via the CHARGE Consortium joint calling cluster file (http://www.chargeconsortium.com/main/exomechip) and quality control of the genotype data was undertaken according to the CHARGE exome chip best practices, described elsewhere.[38]

UK Biobank: The UK Biobank samples were genotyped using the Affymetrix UK BiLEVE and UK Biobank arrays, which include variants which were selected from the same sequencing project as the Illumina Human Exome BeadChip and expected to be polymorphic in UK populations, alongside additional content. The QC and imputation procedure of the UK BiLEVE/UK Biobank genotype data is described elsewhere.[33,39] In brief, thorough sample and genotype QC was undertaken before imputation to a combined 1000G[40] and UK10K Project[41] reference panel. Following imputation, SNPs were excluded if they had imputation INFO score ≤0.5 or minor allele count (MAC)<3.

UKHLS: Genotype calling was performed using the Illumina GenCall software (2). Sample-level quality control (QC) was performed using the following filters: call rate < 98%, autosomal heterozygosity outliers (> 3 SD), gender mismatches, duplicates as established by identity by descent (IBD) analysis (PI_HAT > 0.9), ethnic outliers as determined by combining with 1000 Genomes Project data and carrying out IBD followed by multidimensional scaling. In total, 9,965 samples passed QC. Variant-level QC was performed as follows:

variants were mapped to forward strand of human genome build 37. Variants with Hardy-Weinberg equilibrium $P < 1\times10^{-4}$, a call rate < 98% and poor genotype clustering values (< 0.4) were removed, as well as Y-chromosome and mitochondrial variants.

eQTL Analyses

The sentinel SNPs, and proxies (r^2>0.8) within the newly identified regions were assessed in the following eQTL data sets.

Blood: We searched for blood eQTLs within a publicly available blood eQTL dataset with results from the analysis of 5,311 individuals, imputed to HapMap 2 [42]. Association testing was undertaken both for cis (+/- 250Kb distance between the SNP and the probe midpoint) and trans (distance between the SNP and the probe midpoint >5Mb).

GTEx (all tissues): SNPs were assessed in expression data from samples from the GTEx project [43]. Only cis-eQTLs (+/- 1Mb distance between the SNP and transcription start site were available in the dataset. All available tissues and sample sizes are summarised below.

**Tissue**	**Number of RNASeq and Genotyped samples**	**Number of eGenes**
Muscle - Skeletal	361	7082
Whole Blood	338	6784
Skin - Sun Exposed (Lower leg)	302	8567
Adipose - Subcutaneous	298	8500
Artery - Tibial	285	8056
Thyroid	278	9937
Lung	278	7236
Nerve - Tibial	256	9860
Esophagus - Mucosa	241	7416
Cells - Transformed fibroblasts	272	8760
Skin - Not Sun Exposed (Suprapubic)	196	5491
Esophagus - Muscularis	218	6916
Adipose - Visceral (Omentum)	185	4301
Artery - Aorta	197	6220
Heart - Left Ventricle	190	4417
Breast - Mammary Tissue	183	4140
Colon - Transverse	169	4446
Heart - Atrial Appendage	159	3929
Stomach	170	3438
Testis	157	9009
Pancreas	149	4301
Esophagus - Gastroesophageal Junction	127	2751
Colon - Sigmoid	124	2882
Adrenal Gland	126	3259
Artery - Coronary	118	2363
Brain - Cerebellum	103	4163
Liver	97	1628

Tissue	Number of RNASeq and Genotyped samples	Number of eGenes
Cells - EBV-transformed lymphocytes	114	2957
Brain - Caudate (basal ganglia)	100	2447
Brain - Cortex	96	2567
Brain - Nucleus accumbens (basal ganglia)	93	2019
Brain - Frontal Cortex (BA9)	92	2009
Prostate	87	1462
Brain - Cerebellar Hemisphere	89	3251
Spleen	89	2754
Pituitary	87	2160
Brain - Putamen (basal ganglia)	82	1588
Ovary	85	1583
Brain - Hypothalamus	81	1157
Vagina	79	840
Brain - Hippocampus	81	1134
Small Intestine - Terminal Ileum	77	1356
Brain - Anterior cingulate cortex (BA24)	72	1212
Uterus	70	917

Lung resection cohort: Selected SNPs were assessed in a lung eQTL resource based on lung tissues of 1,111 individuals. The descriptions of the lung eQTL dataset and subject demographics have been published previously [44–46]. Briefly, non-tumor lung tissues were collected from patients who underwent lung resection surgery at three participating sites: Laval University (Quebec City, Canada), University of Groningen (Groningen, The Netherlands), and University of British Columbia (Vancouver, Canada). Whole-genome gene expression and genotyping data were obtained from these specimens. Gene expression profiling was performed using an Affymetrix custom array (GPL10379) testing 51,627 non-control probe sets and normalized using RMA[47]. Genotyping was performed using the Illumina Human1M-Duo BeadChip array. Genotype imputation was undertaken using the 1000 Genomes Project [40] reference panel. Following standard microarray and genotyping quality controls, 1111 patients were available including 409 from Laval, 363 from Groningen, and 339 from UBC. Lung eQTLs were identified to associate with mRNA expression in either cis (within 1 Mb of transcript start site) or in trans (all other eQTLs).

Analysis: Look-ups in the lung resection cohort, blood and GTEx (all tissues) datasets were undertaken for the 7 sentinel SNPs identified in the combined SpiroMeta-CHARGE analysis, and all proxies with $r^2>0.8$. For all datasets, eQTL associations were identified as significant at an FDR of 5% (or q-value<0.05). For any gene identified in the eQTL look-up, the most significantly associated eSNP for each gene was identified. All genes for which the sentinel lung function SNP and the top eSNP were in strong linkage disequilibrium ($r^2>0.9$) were further highlighted (i.e. where there was co-localisation of the lung function associated SNP and the gene expression associated SNP).

Supplementary Figures

Supplementary Figure1

Quantile-quantile (QQ) and Manhattan plots for consortium-wide analyses (CHARGE, top; SpiroMeta, middle), and the combined meta-analysis (bottom). Results shown are for the analysis of all individuals (never and ever smokers combined, and both European and African ancestry), for three lung function traits: A. FEV_1, B. FVC C. FEV_1/FVC.

A. FEV_1

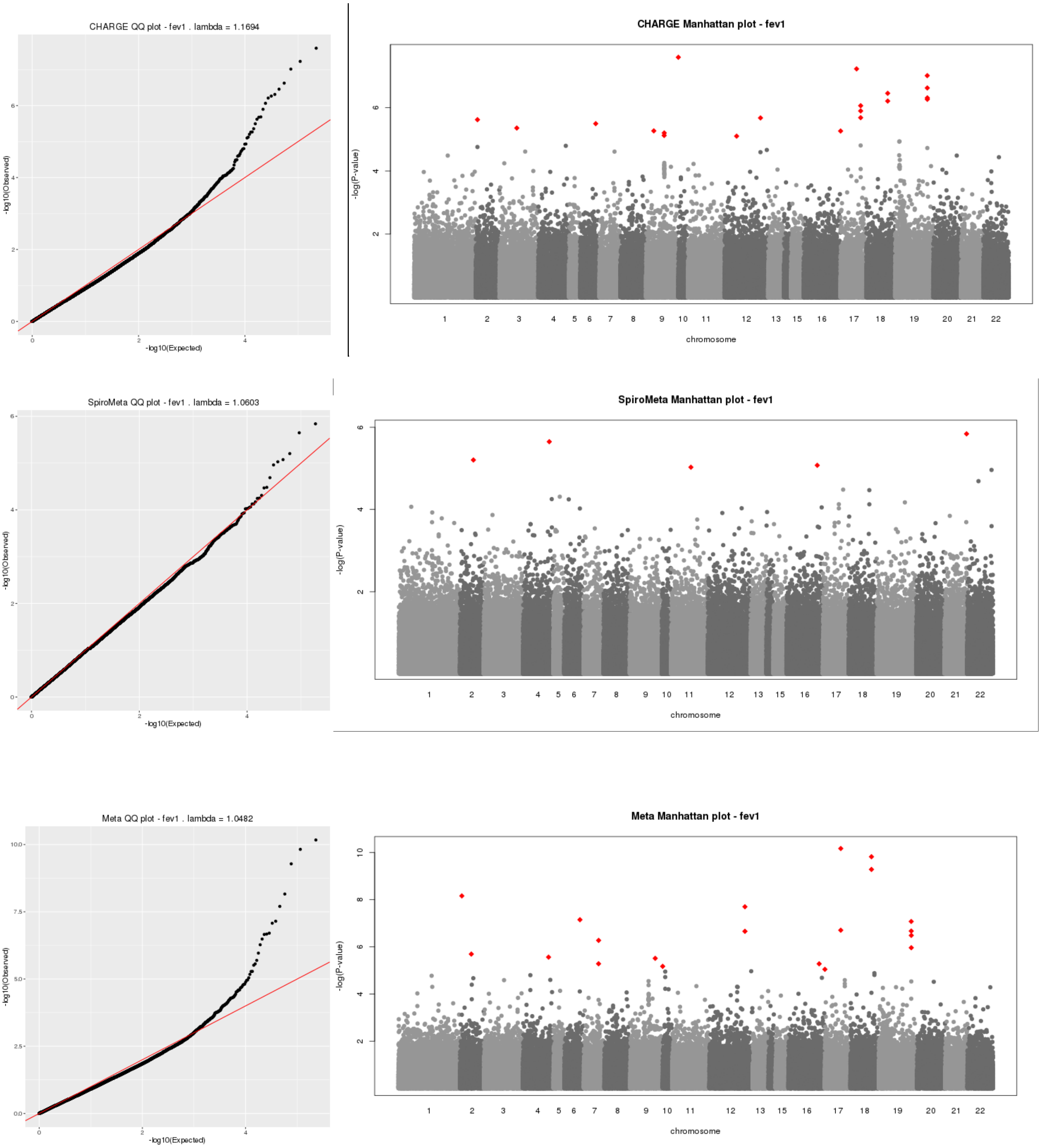

B. FVC

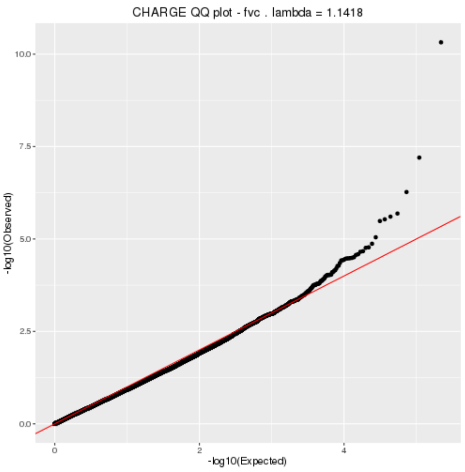
CHARGE QQ plot - fvc . lambda = 1.1418
-log10(Observed)
-log10(Expected)

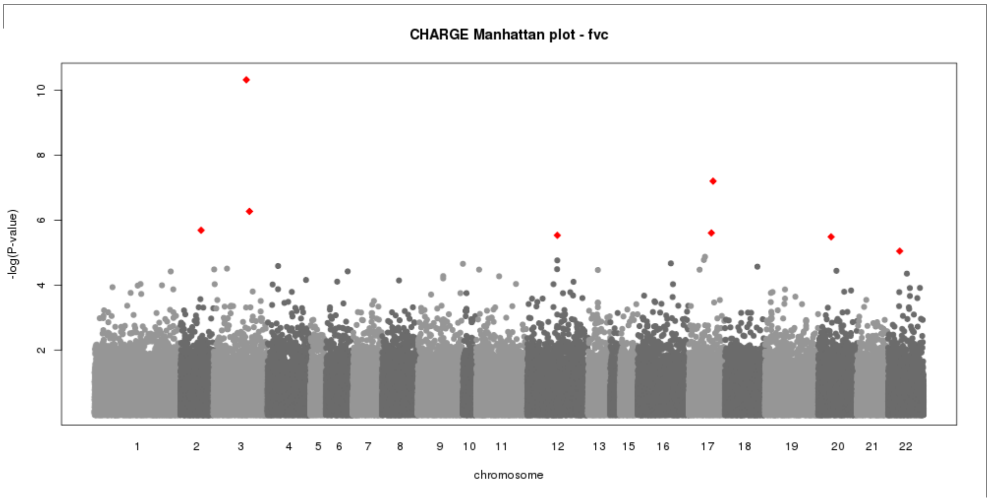
CHARGE Manhattan plot - fvc
-log(P-value)
1 2 3 4 5 6 7 8 9 10 11 12 13 15 16 17 18 19 20 21 22
chromosome

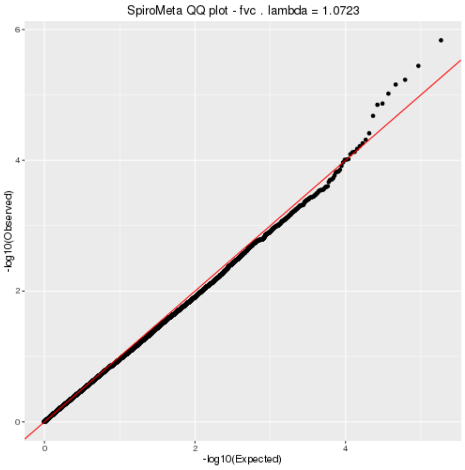
SpiroMeta QQ plot - fvc . lambda = 1.0723
-log10(Observed)
-log10(Expected)

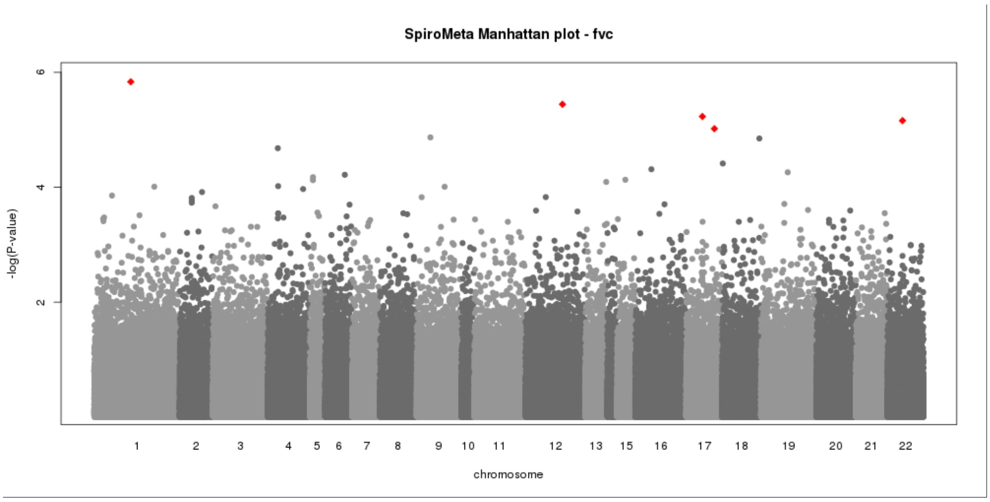
SpiroMeta Manhattan plot - fvc
-log(P-value)
1 2 3 4 5 6 7 8 9 10 11 12 13 15 16 17 18 19 20 21 22
chromosome

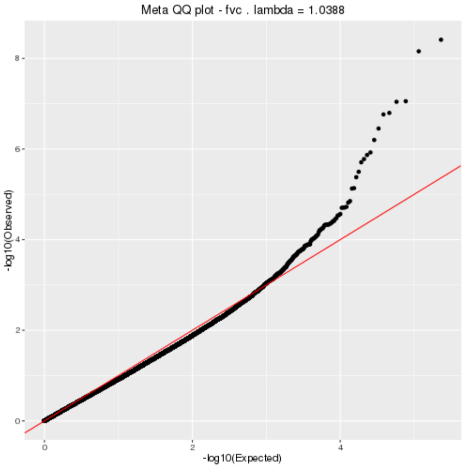
Meta QQ plot - fvc . lambda = 1.0388
-log10(Observed)
-log10(Expected)

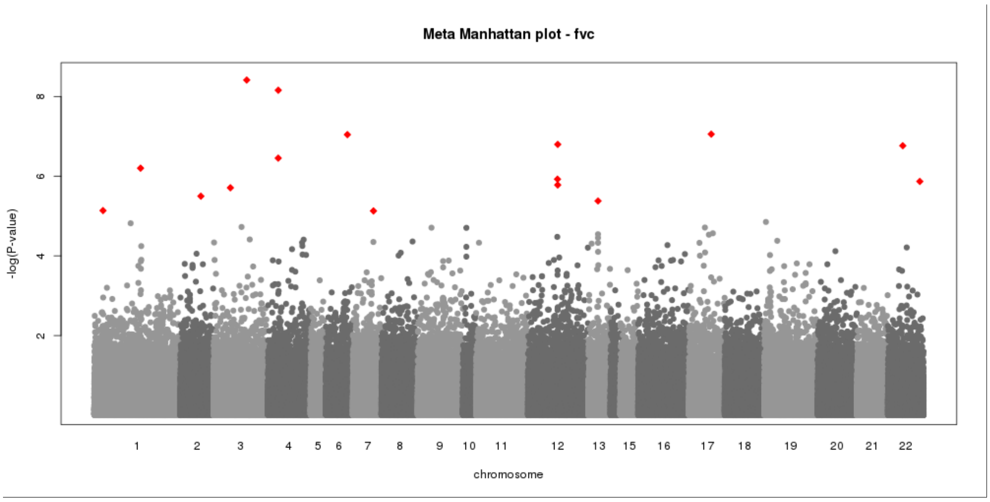
Meta Manhattan plot - fvc
-log(P-value)
1 2 3 4 5 6 7 8 9 10 11 12 13 15 16 17 18 19 20 21 22
chromosome

C. FEV$_1$/FVC

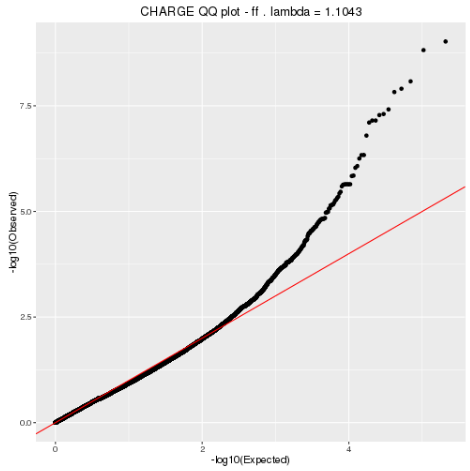
CHARGE QQ plot - ff . lambda = 1.1043
-log10(Observed)
-log10(Expected)

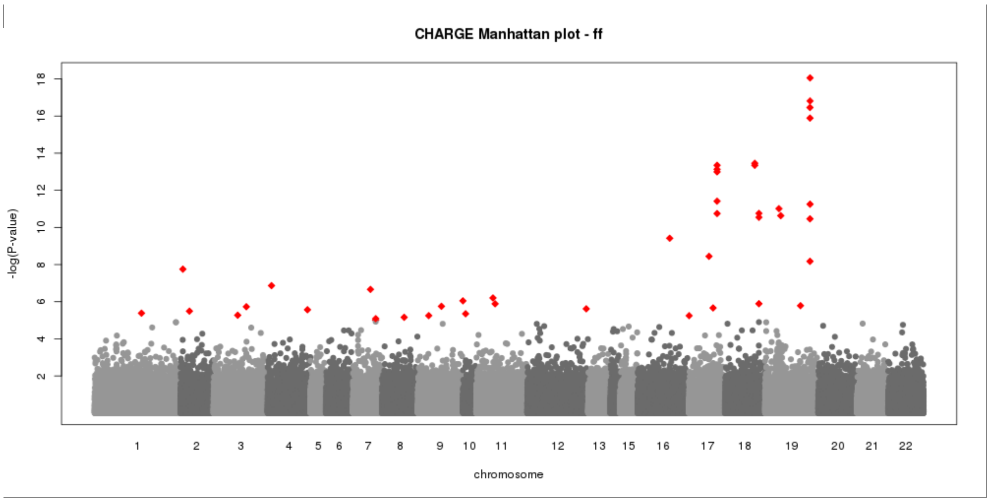
CHARGE Manhattan plot - ff
-log(P-value)
chromosome

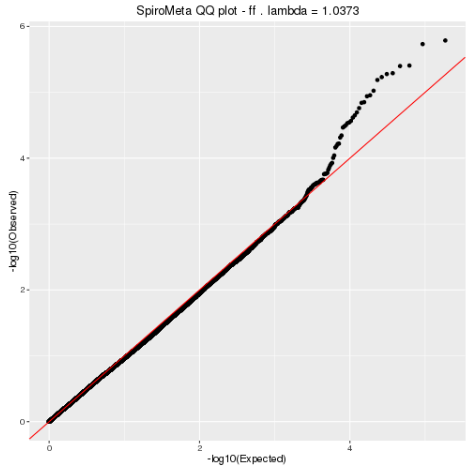
SpiroMeta QQ plot - ff . lambda = 1.0373
-log10(Observed)
-log10(Expected)

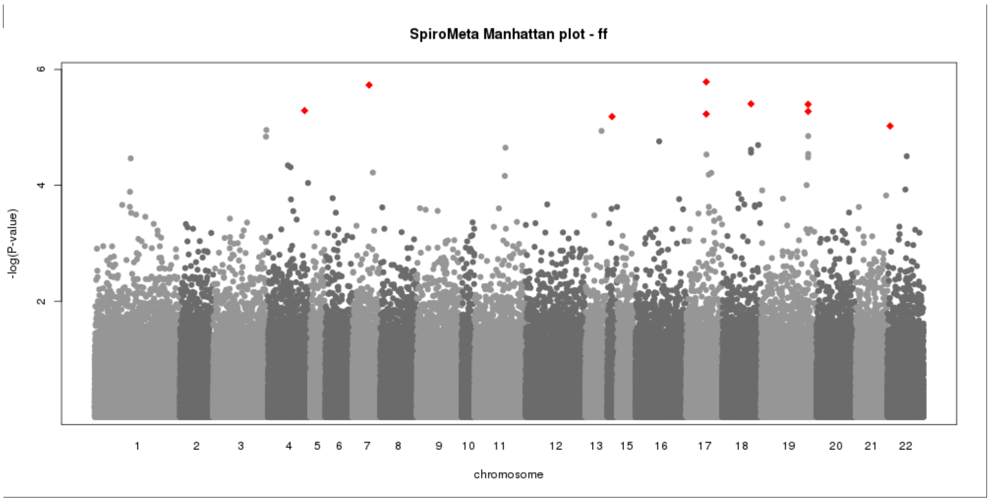
SpiroMeta Manhattan plot - ff
-log(P-value)
chromosome

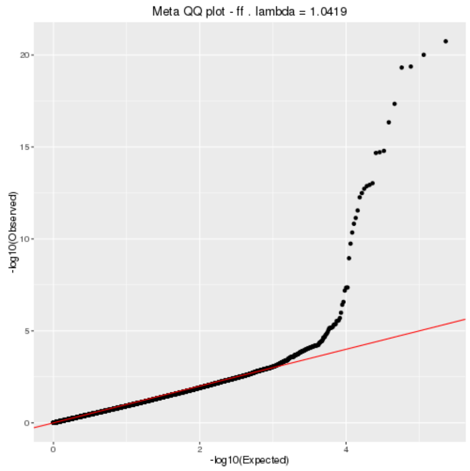
Meta QQ plot - ff . lambda = 1.0419
-log10(Observed)
-log10(Expected)

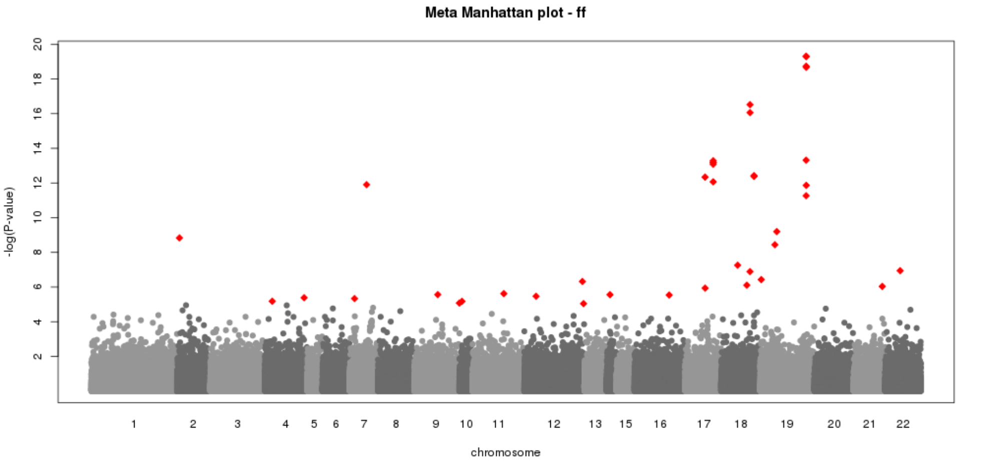
Meta Manhattan plot - ff
-log(P-value)
chromosome

Supplementary Figure 2: Region Plots for novel loci.

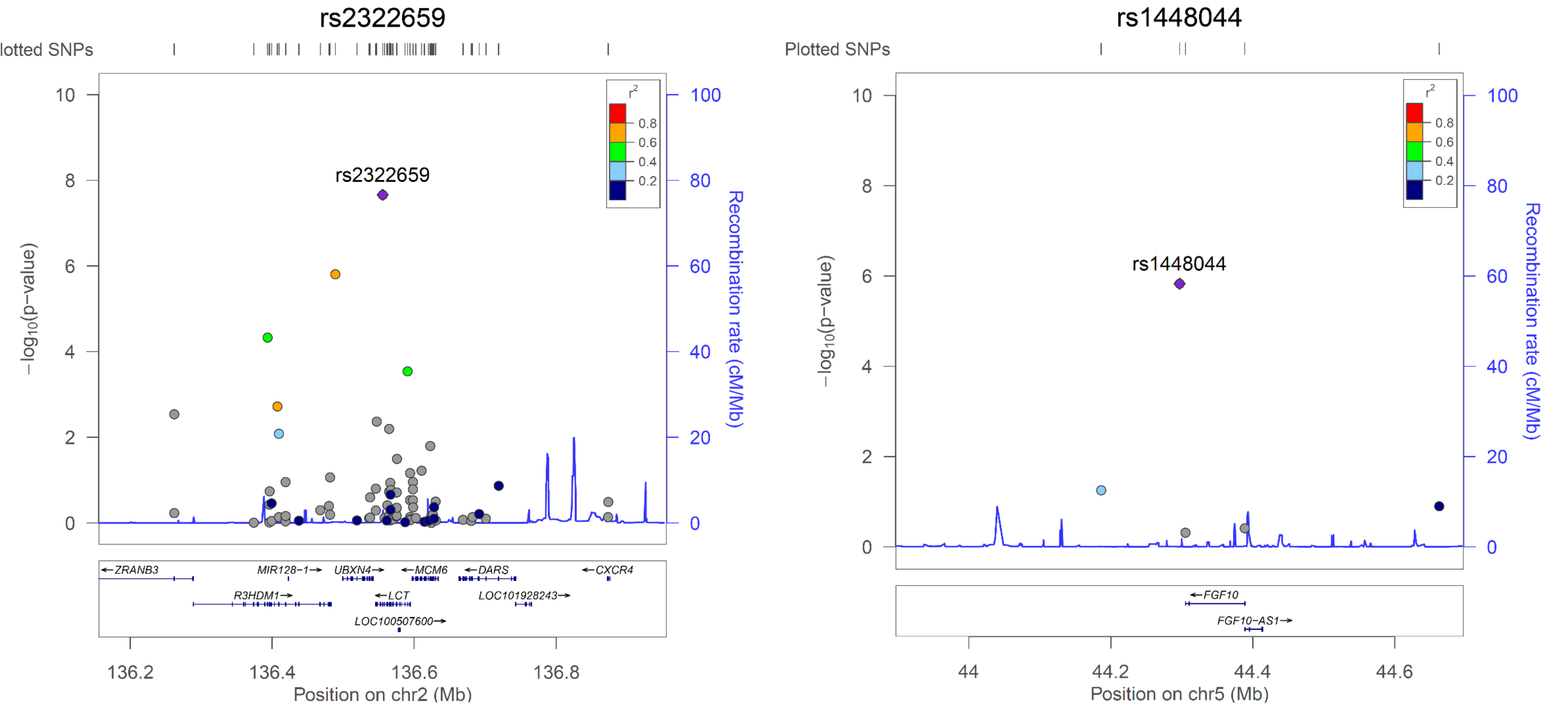

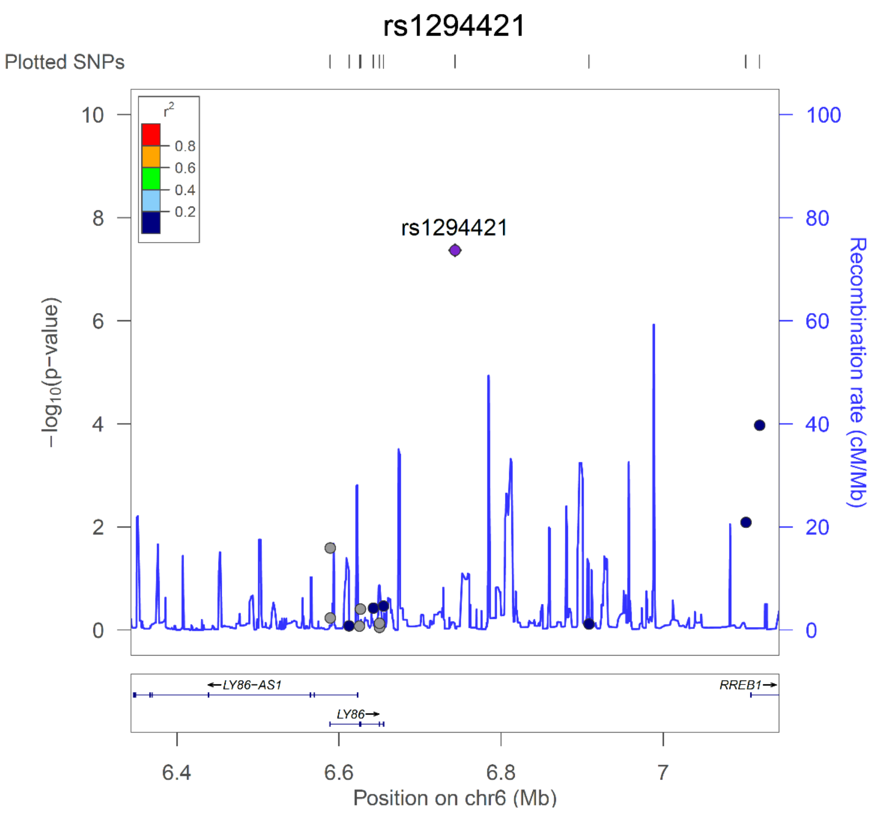
rs1294421
Plotted SNPs
−log10(p−value)
Recombination rate (cM/Mb)
r2
0.8
0.6
0.4
0.2
rs1294421
LY86−AS1
LY86
RREB1
Position on chr6 (Mb)

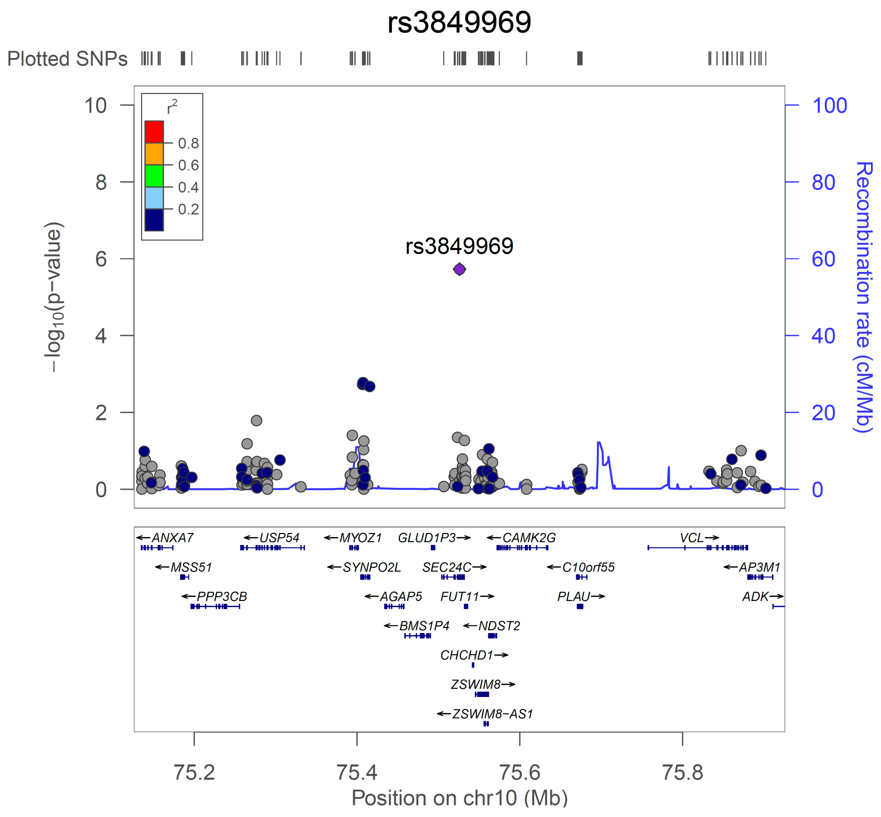
rs3849969
Plotted SNPs
−log10(p−value)
Recombination rate (cM/Mb)
r2
0.8
0.6
0.4
0.2
rs3849969
ANXA7
USP54
MYOZ1
GLUD1P3
CAMK2G
VCL
MSS51
SYNPO2L
SEC24C
C10orf55
AP3M1
PPP3CB
AGAP5
FUT11
PLAU
ADK
BMS1P4
NDST2
CHCHD1
ZSWIM8
ZSWIM8−AS1
Position on chr10 (Mb)

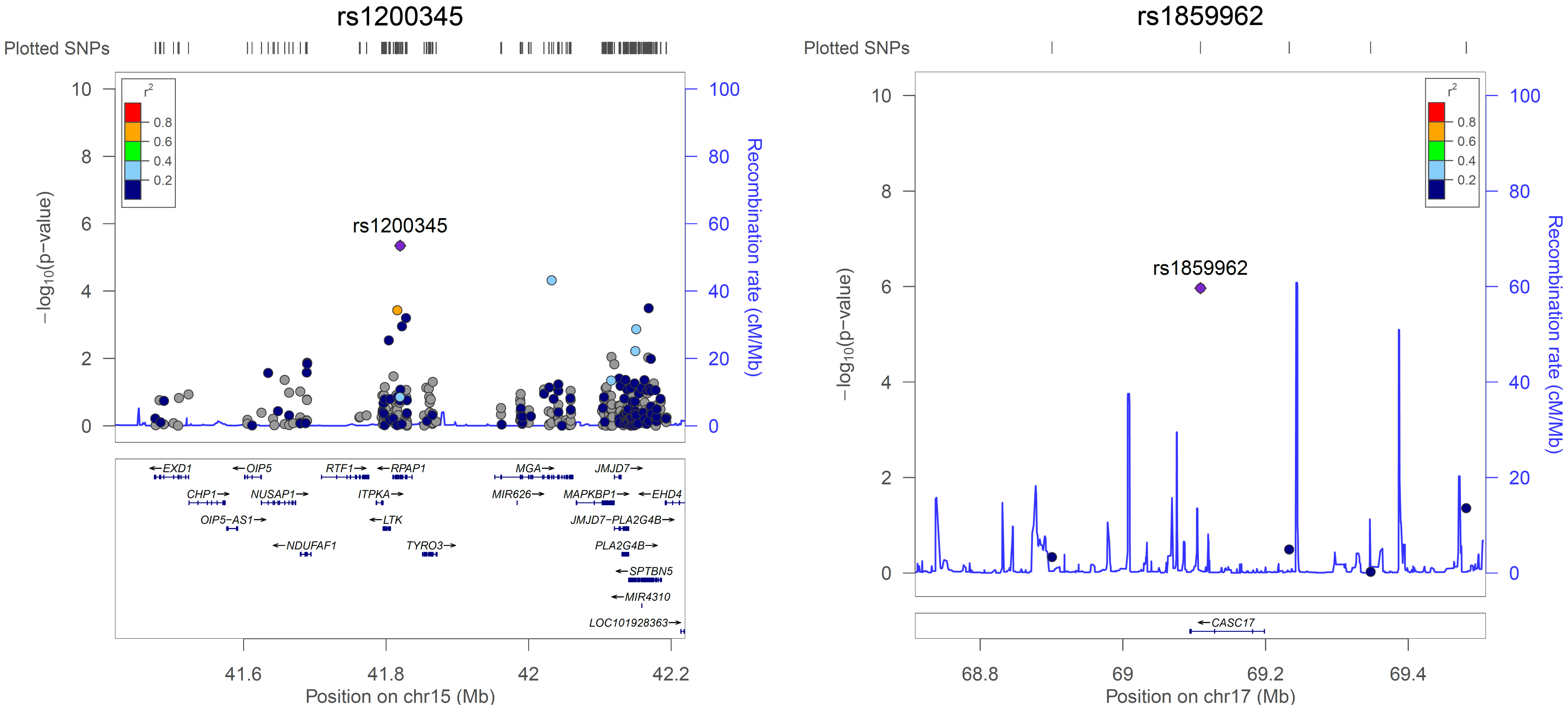
rs1200345
Plotted SNPs
rs1200345
r²
0.8
0.6
0.4
0.2
−log10(p−value)
Recombination rate (cM/Mb)
EXD1
OIP5
RTF1
RPAP1
MGA
JMJD7
CHP1
NUSAP1
ITPKA
MIR626
MAPKBP1
EHD4
OIP5−AS1
LTK
JMJD7−PLA2G4B
NDUFAF1
TYRO3
PLA2G4B
SPTBN5
MIR4310
LOC101928363
41.6
41.8
42
42.2
Position on chr15 (Mb)
rs1859962
Plotted SNPs
rs1859962
−log10(p−value)
Recombination rate (cM/Mb)
CASC17
68.8
69
69.2
69.4
Position on chr17 (Mb)

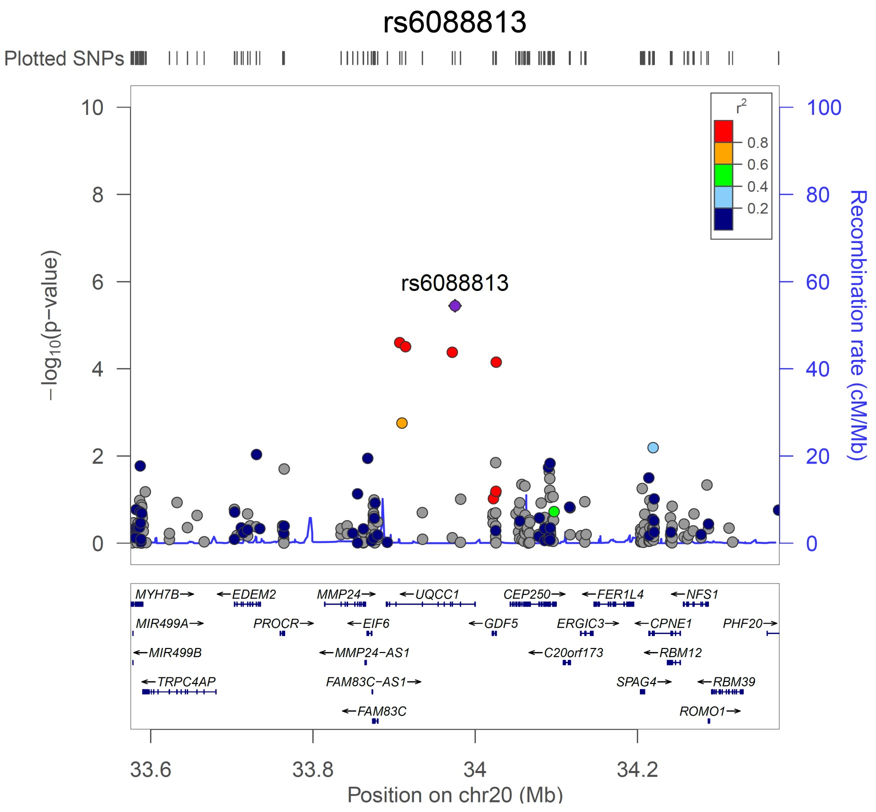

rs6088813
Plotted SNPs
−log10(p−value)
Recombination rate (cM/Mb)
r2
0.8
0.6
0.4
0.2
rs6088813
MYH7B
EDEM2
MMP24
UQCC1
CEP250
FER1L4
NFS1
MIR499A
PROCR
EIF6
GDF5
ERGIC3
CPNE1
PHF20
MIR499B
MMP24−AS1
C20orf173
RBM12
TRPC4AP
FAM83C−AS1
SPAG4
RBM39
FAM83C
ROMO1
33.6
33.8
34
34.2
Position on chr20 (Mb)

Supplementary Figure 3: Forest plots of novel loci.

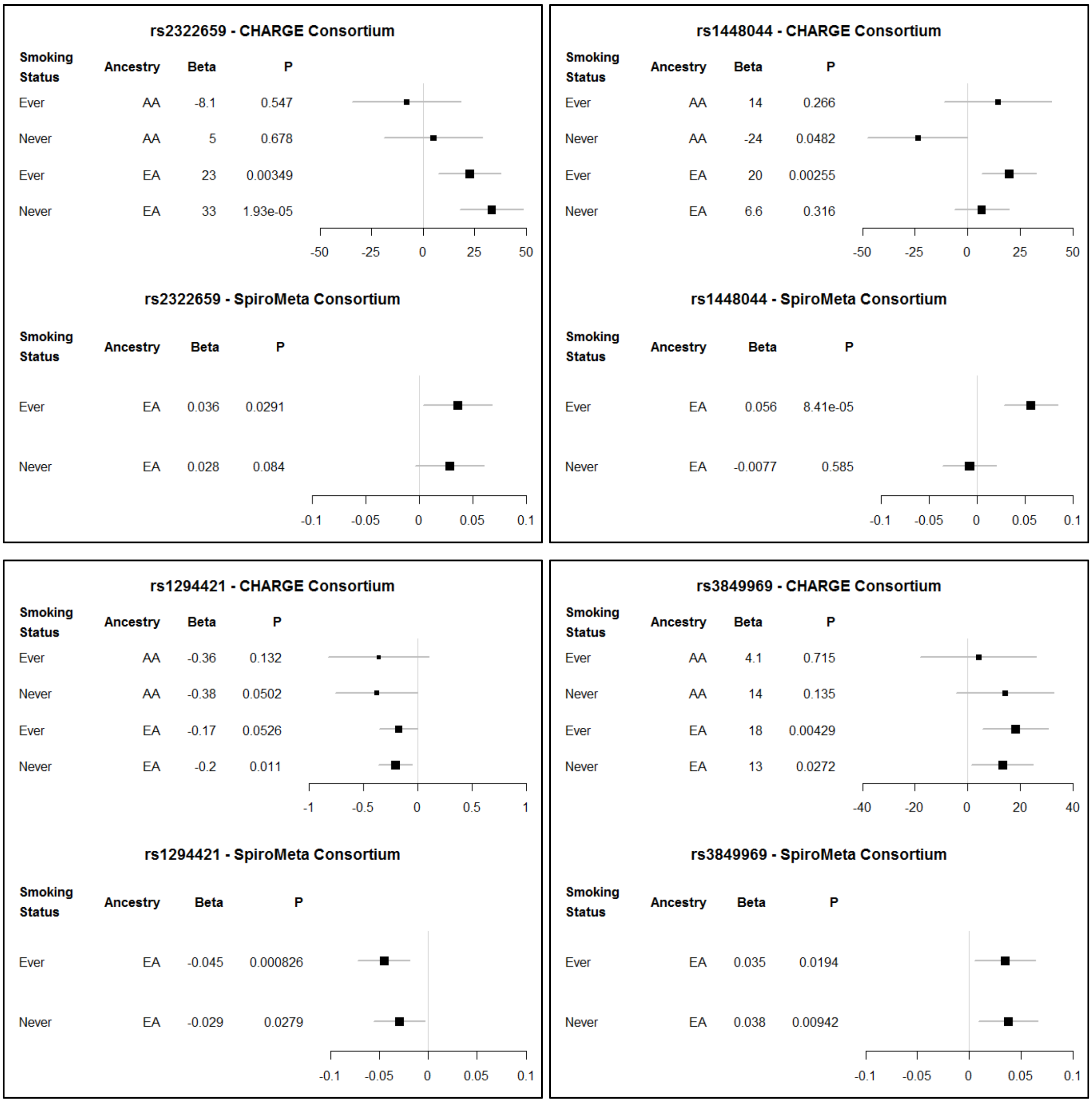

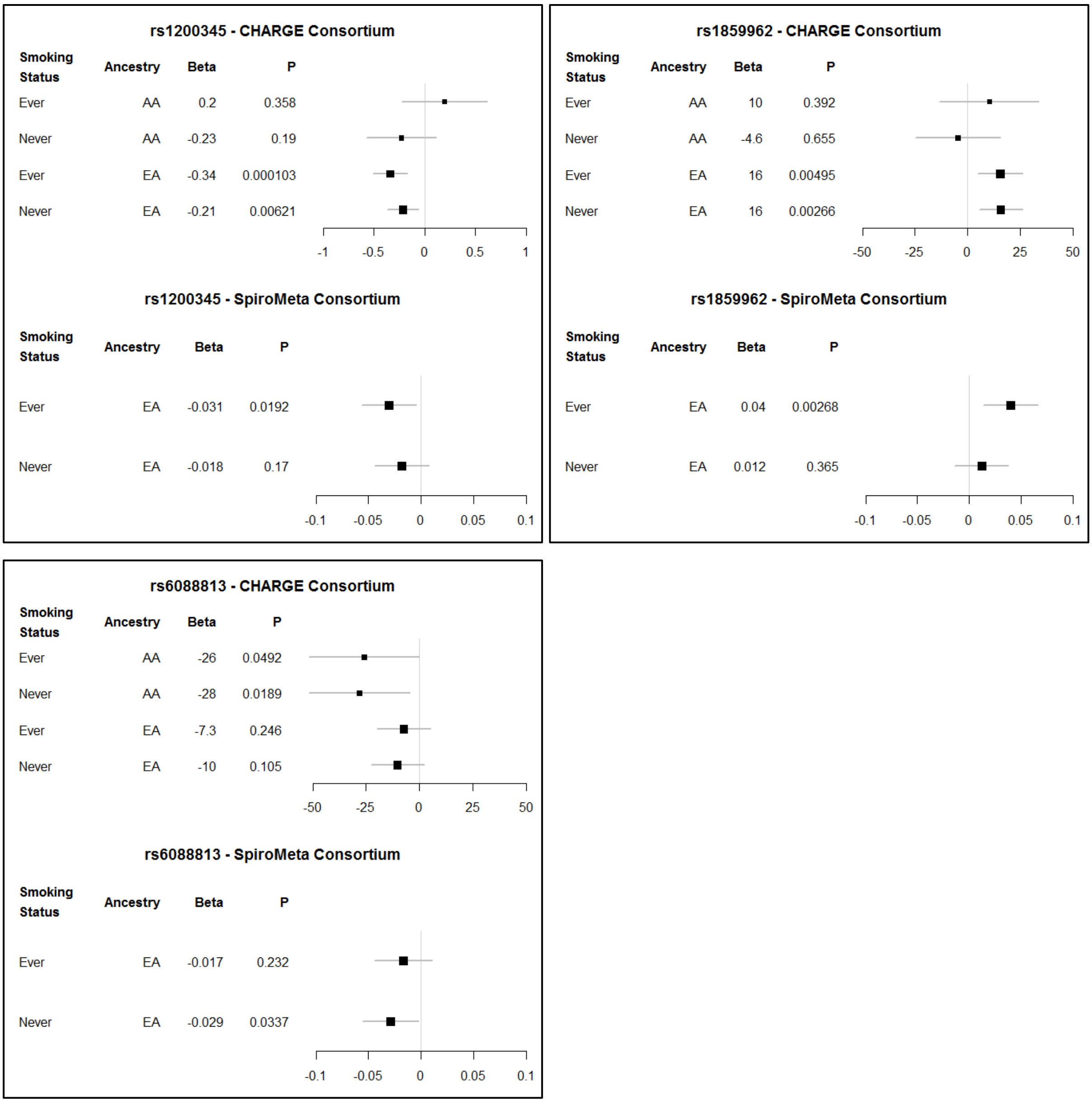
rs1200345 - CHARGE Consortium
Smoking Status
Ancestry
Beta
P
Ever AA 0.2 0.358
Never AA -0.23 0.19
Ever EA -0.34 0.000103
Never EA -0.21 0.00621
-1 -0.5 0 0.5 1
rs1200345 - SpiroMeta Consortium
Smoking Status
Ancestry
Beta
P
Ever EA -0.031 0.0192
Never EA -0.018 0.17
-0.1 -0.05 0 0.05 0.1
rs1859962 - CHARGE Consortium
Smoking Status
Ancestry
Beta
P
Ever AA 10 0.392
Never AA -4.6 0.655
Ever EA 16 0.00495
Never EA 16 0.00266
-50 -25 0 25 50
rs1859962 - SpiroMeta Consortium
Smoking Status
Ancestry
Beta
P
Ever EA 0.04 0.00268
Never EA 0.012 0.365
-0.1 -0.05 0 0.05 0.1
rs6088813 - CHARGE Consortium
Smoking Status
Ancestry
Beta
P
Ever AA -26 0.0492
Never AA -28 0.0189
Ever EA -7.3 0.246
Never EA -10 0.105
-50 -25 0 25 50
rs6088813 - SpiroMeta Consortium
Smoking Status
Ancestry
Beta
P
Ever EA -0.017 0.232
Never EA -0.029 0.0337
-0.1 -0.05 0 0.05 0.1

Supplementary Figure 4: Trait Transformation Sensitivity Analysis.

We have repeated the analysis for the six reported SNPs (the *LCT* SNP was not available in UK Biobank), transforming the phenotypes, and then adjusting for all covariates (including PCs) during the SNP-trait association test. For comparison, we have done this for all six SNPs with all three traits. Comparisons of these two analyses (not adjusted prior to transformation vs with adjustment prior to transformation) are shown here. For each SNP, the P-value comparison is highlighted for the trait we report the association with, and the dashed lines indicate the Bonferroni corrected significance threshold for independent replication ($P<1{\cdot}47\times10^{-3}$). Whilst there is a difference in the P-values for some SNP-trait combinations, (more significant P-values in the analysis with covariate adjustment prior to transformation for 5 of the 6 SNPs), the SNPs all meet the replication P-value threshold in both analyses.

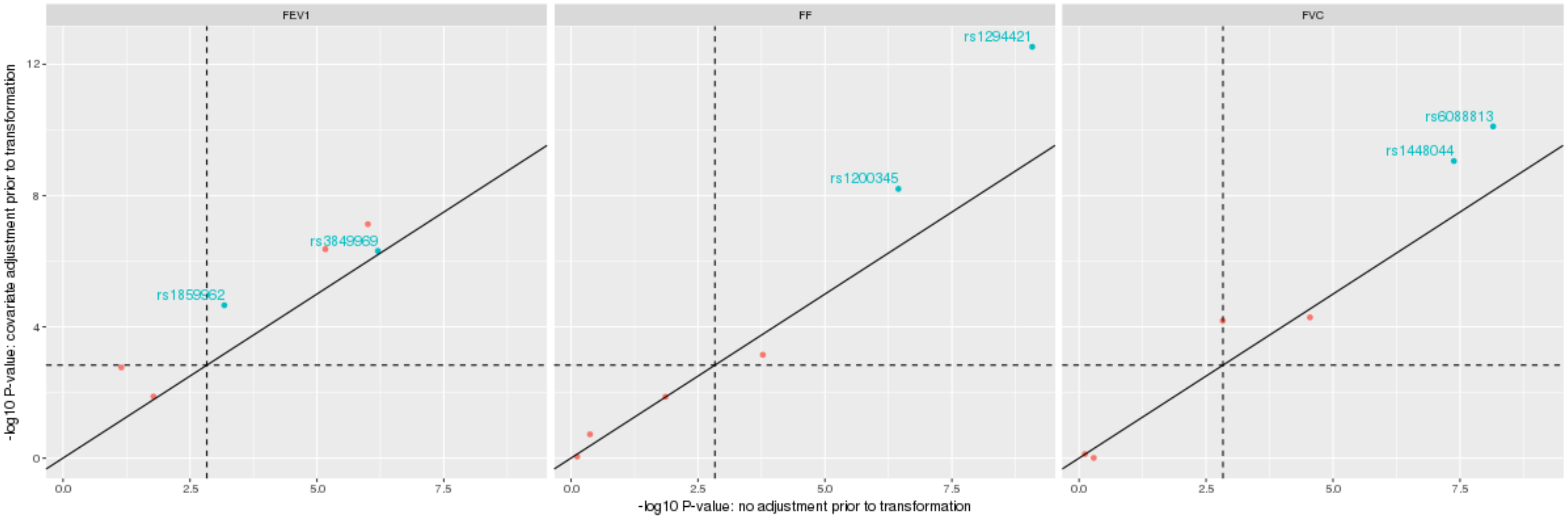

Supplementary Tables

Supplementary Table 1: Details of study specific genotyping platform, genotype calling procedure and software.

**Discovery Cohorts**			
**Study Name**	**Genotyping Platform**	**Calling algorithm(s)**	**Study-level software**
B58C	Illumina Human Exome BeadChip v1	Gencall + zCall	RAREMETALWORKER
GS:SFHS	Illumina Human Exome BeadChip v1	CHARGE	RAREMETALWORKER
KORA F4	Illumina Human Exome BeadChip v1	Gencall + zCall	RAREMETALWORKER
KORCULA	Illumina Human Exome BeadChip v1	CHARGE	RAREMETALWORKER
LBC 1936	Illumina Human Exome BeadChip v1	CHARGE	rvtests
SHIP	Illumina Human Exome BeadChip v1	Gencall + zCall	RAREMETALWORKER
NSPHS	Illumina Human Exome BeadChip v1	Gencall + zCall	RAREMETALWORKER
PIVUS	Illumina Human Exome BeadChip v1	Gencall + zCall	RAREMETALWORKER
SAPALDIA	Illumina Human Exome BeadChip v1	Gencall + zCall	RAREMETALWORKER
YFS	Illumina Human Exome BeadChip v1	Gencall	rvtests
FIN	Illumina Human Exome BeadChip v1	Gencall + zCall	RAREMETALWORKER
AGES	Illumina Human Exome BeadChip v1	CHARGE	Seqmeta
ARIC	Illumina Human Exome BeadChip v1	CHARGE	Seqmeta
CHS	Illumina Human Exome BeadChip v1	CHARGE	Seqmeta
FAMHS	Illumina Human Exome BeadChip v1	CHARGE	Seqmeta
FHS	Illumina Human Exome BeadChip v1	CHARGE	Seqmeta
HABC	Illumina Human Exome BeadChip v1	CHARGE	Seqmeta
Health2006	Illumina Human Exome BeadChip v1	CHARGE	Seqmeta
Health2008	Illumina Human Exome BeadChip v1	CHARGE	Seqmeta
Inter99np	Illumina Human Exome BeadChip v1	CHARGE	Seqmeta
Inter99p	Illumina Human Exome BeadChip v1	CHARGE	Seqmeta
JHS	Illumina Human Exome BeadChip v1	CHARGE	Seqmeta
MESA	Illumina Human Exome BeadChip v1	CHARGE	Seqmeta
RS	Illumina Human Exome BeadChip v1	CHARGE	Seqmeta
**Replication Cohorts**			
**Study Name**	**Genotyping Platform**	**Calling algorithm(s)**	**Study-level software**
UK Biobank	Affymetrix Axiom UK BiLEVE array and Affymetrix Axiom UK Biobank array	Axiom® GT1 algorithm (Affymetrix Power Tools v1.15.1)	Imputation: SHAPEIT and IMPUTE2. Association Testing: SNPTEST (single variant associations, imputed data); RAREMETALWORKER (gene-based associations, genotyped SNPs only).
UKHLS	Illumina Infinium HumanCoreExome-12 v1.0BeadChip	GenCall	RAREMETALWORKER
NEO	Illumina Infinium HumanCoreExome-12 v1.0BeadChip	CHARGE	Seqmeta

Supplementary Table 2: Association results for all SNPs identified in single variant association discovery analyses ($P<10^{-4}$). Only variants in novel loci shown, and results presented for the trait, smoking and ancestry combination for which the strongest association was identified. Variants were followed up for the trait and smoking subset for which they were most significantly associated (only European Ancestry samples available). Chromosome (CHROM) and position (POS) in build 37 are given for each SNP. For the SpiroMeta Consortium, beta values reflect effect-size estimates on an inverse-normal transformed scale after adjustments for age, age^2, sex, height and ancestry principal components. For the CHARGE Consortium, beta values represent untransformed trait effect estimates (ml or ratio), after adjustment for former smoking, current smoking and pack-years of smoking, age, age^2, sex, height , $height^2$, centre/cohort, principle components and weight (FVC only).

							**Consortium level Discovery Analysis**													
							**CHARGE Consortium**		**SpiroMeta Consortium**		**Discovery Meta-analysis**				**Overall Replication**				**Combined Discovery + Replication Result**	
**dbSNPID**	**Chr:Pos**	**effect/ noneffect allele**	**(nearest) gene**	**Trait**	**Smoking subset (ever /never smokers / all)**	**Ancestry subset (both [EA+AA] / EA only)**	**Beta**	**P-value**	**Beta**	**P-value**	**N**	**Effect Allele Frequency (EAF)**	**Z-score**	**P-value**	**N**	**EAF**	**Z-score**	**P-value**	**Z-score**	**P-value**
rs61744357	1:16134072	T/C	*UQCRHL*	FVC	all	both	-17.522	1.04E-3	-0.070	3.58E-4	57363	15.092%	-4.516	6.29E-6	104113	11.641%	0.455	0.6489	-2.326	2.00E-2
rs41273537	1:150429944	G/A	*RPRD2*	FEV1	never	both	-30.677	1.39E-4	-0.072	2.96E-4	28519	10.789%	-5.197	2.03E-7	47087	12.019%	-1.754	0.0795	-4.552	5.32E-6
rs202079239	1:154548277	G/C	*CHRNB2*	FEV1	ever	EA	909.281	6.21E-5	0.998	3.69E-3	27269	0.026%	4.885	1.03E-6	4506	0.022%	1.437	0.1507	5.066	4.05E-7
rs145942857	2:45801809	C/T	*SRBD1*	FEV1/ FVC	all	EA	-9.948	1.80E-5	-0.604	2.69E-2	60381	0.021%	-4.650	3.32E-6	7443	0.034%	0.765	0.4442	-4.062	4.87E-5
rs2322659	2:136555659	T/C	*LCT*	FVC	all	EA	27.341	2.82E-7	0.032	7.60E-3	55591	23.560%	5.597	2.18E-8	12899	20.134%	2.286	0.0223	6.024	**1.70E-9**
rs147184138	3:25833094	G/C	*OXSM*	FVC	ever	EA	321.888	7.05E-3	0.903	7.53E-5	27268	0.077%	4.609	4.04E-6	4506	0.078%	0.253	0.8004	4.365	1.27E-5
rs201776558	3:54922021	A/G	*CACNA2D3*	FVC	ever	EA	-1196.294	3.15E-4	-1.393	2.39E-3	27268	0.015%	-4.698	2.63E-6	4504	0.000%	0.000	1.0000	-4.352	1.35E-5
rs150210060	3:113753937	A/G	*KIAA1407*	FVC	ever	EA	135.506	4.08E-5	0.156	2.19E-2	27268	0.942%	4.592	4.38E-6	64472	1.102%	-0.333	0.7394	2.225	2.61E-2
rs35706839	3:182733255	T/C	*MCCC1*	FVC	all	EA	1159.392	5.02E-5	2.054	5.16E-3	60394	0.005%	4.861	1.17E-6	7441	0.000%	0.000	1.0000	4.534	5.78E-6
rs199529766	3:185783622	T/C	*ETV5*	FEV1	ever	EA	-1346.179	2.41E-4	-2.768	5.77E-3	27269	0.006%	-4.542	5.57E-6	4506	0.011%	0.458	0.6472	-4.035	5.45E-5
rs202225480	4:10502971	G/A	*CLNK*	FEV1	ever	EA	1163.190	6.29E-4	1.528	1.02E-3	27269	0.013%	4.690	2.74E-6	4506	0.000%	0.000	1.0000	4.344	1.40E-5
rs1448044	5:44296986	A/G	*FGF10*	FVC	ever	both	18.628	2.02E-3	0.056	8.48E-5	30966	35.637%	4.813	1.49E-6	64400	32.646%	4.805	**1.55E-6**	6.691	**2.22E-11**
rs1294421	6:6743149	T/G	*LY86*	FEV1/ FVC	all	both	-0.222	3.83E-5	-0.037	1.22E-4	68099	36.786%	-5.479	4.27E-8	111556	39.273%	-8.171	**3.06E-16**	-9.815	**9.74E-23**
rs200718368	6:28053978	C/T	*ZNF165*	FEV1/ FVC	never	both	-14.186	1.55E-6	-0.685	4.08E-2	28504	0.025%	-4.953	7.29E-7	2940	0.000%	0.000	1	-4.603	4.17E-6
rs4713479	6:31688799	T/C	*LY6G6C*	FEV1/ FVC	ever	both	-0.799	1.24E-4	-0.107	9.96E-3	30967	4.250%	-4.551	5.33E-6	60910	2.974%	-1.710	0.0873	-4.035	5.47E-5

							Consortium level Discovery Analysis													
							CHARGE Consortium		SpiroMeta Consortium		Discovery Meta-analysis				Overall Replication				Combined Discovery + Replication Result	
dbSNPID	Chr:Pos	effect/ noneffect allele	(nearest) gene	Trait	Smoking subset (ever /never smokers / all)	Ancestry subset (both [EA+AA] / EA only)	Beta	P-value	Beta	P-value	N	Effect Allele Frequency (EAF)	Z-score	P-value	N	EAF	Z-score	P-value	Z-score	P-value
rs200840680	7:138978128	C/T	*UBN2*	FEV1/ FVC	never	both	-10.334	5.83E-5	-1.043	2.25E-2	28504	0.018%	-4.510	6.49E-6	2937	0.000%	0.000	1	-4.191	2.78E-5
rs143386950	8:69033248	T/C	*PREX2*	FEV1/ FVC	ever	both	-47.934	2.64E-5	-2.187	2.90E-2	30967	0.003%	-4.598	4.26E-6	4506	0.000%	0.000	1	-4.296	1.74E-5
rs201821244	8:144808545	G/T	*FAM83H*	FEV1/ FVC	all	EA	-29.719	3.90E-5	-2.911	4.16E-3	60381	0.002%	-4.914	8.92E-7	7441	0.000%	0.000	1	-4.584	4.56E-6
rs189491808	9:79321371	A/G	*PRUNE2*	FEV1/ FVC	ever	EA	-7.332	2.15E-4	-0.560	9.40E-3	27260	0.073%	-4.448	8.65E-6	8068	0.087%	-0.997	0.3186	-4.384	1.16E-5
rs3849969	10:75525999	T/C	*SEC24C*	FEV1	all	both	13.095	3.83E-4	0.036	6.99E-4	68116	29.402%	4.767	1.87E-6	111556	27.835%	5.042	**4.60E-7**	6.906	**4.99E-12**
rs148545029	11:102589238	A/G	*MMP8*	FEV1	never	both	-1168.532	1.88E-6	-0.766	4.46E-2	28519	0.018%	-4.893	9.94E-7	2940	0.078%	0.549	0.5831	-4.344	1.40E-5
rs149662873	12:7015117	G/A	*LRRC23*	FEV1/ FVC	never	both	-19.289	1.17E-6	-1.759	1.29E-2	28504	0.007%	-5.279	1.30E-7	2940	0.011%	1.036	0.3001	-4.523	6.11E-6
rs142171275	14:57741119	G/A	*MUDENG*	FVC	never	both	-575.432	4.11E-4	-1.174	2.14E-3	28515	0.030%	-4.612	3.99E-6	2940	0.011%	-0.431	0.6665	-4.445	8.78E-6
rs1200345	15:41819716	C/T	*RPAP1*	FEV1/ FVC	all	EA	-0.216	9.02E-5	-0.025	9.92E-3	60381	48.785%	-4.586	4.51E-6	111556	49.465%	-5.725	**1.03E-8**	-7.329	**2.33E-13**
rs141062694	16:31049855	T/C	*STX4*	FEV1/ FVC	ever	both	-18.063	2.00E-5	-1.661	2.13E-2	30967	0.011%	-4.718	2.38E-6	4506	0.011%	0.375	0.7073	-4.274	1.92E-5
rs142933706	17:3921203	C/G	*ZZEF1*	FVC	never	both	-1961.144	4.36E-5	-2.288	2.24E-2	28515	0.004%	-4.531	5.86E-6	2940	0.000%	0.000	1	-4.211	2.54E-5
rs144838552	17:7838371	T/C	*CNTROB*	FEV1/ FVC	ever	EA	-34.085	2.49E-6	-0.944	3.73E-2	26835	0.011%	-4.887	1.02E-6	60910	0.001%	-1.358	0.1743	-3.834	1.26E-4
rs139231634	17:51901858	A/C	*KIF2B*	FEV1/ FVC	ever	both	-3.523	2.84E-5	-0.254	4.62E-2	30967	0.252%	-4.471	7.80E-6	60910	0.231%	-1.532	0.1255	-3.843	1.22E-4
rs1859962	17:69108753	G/T	*CASC17*	FEV1	all	EA	15.389	2.75E-5	0.026	7.50E-3	60395	48.171%	4.876	1.08E-6	111554	46.464%	4.612	**3.99E-6**	6.600	**4.10E-11**
rs61739354	17:78039309	T/G	*CCDC40*	FEV1/ FVC	never	EA	21.157	1.89E-4	2.660	9.40E-3	24483	0.004%	4.441	8.94E-6	2940	0.000%	0.000	1	4.082	4.47E-5
rs6088813	20:33975181	C/A	*UQCC1*	FVC	all	both	-16.157	3.44E-5	-0.023	2.29E-2	68115	41.441%	-4.634	3.58E-6	111556	36.898%	-7.688	**1.50E-14**	-8.915	**4.90E-19**
rs6004919	22:26455216	T/C	*NA*	FVC	ever	both	-37.193	4.82E-5	-0.052	3.31E-2	30966	15.546%	-4.488	7.19E-6	8068	8.193%	-1.342	0.1795	-4.608	4.07E-6
rs112306225	22:37261097	A/C	*NCF4*	FEV1/ FVC	ever	EA	-5.084	9.00E-6	-0.347	1.18E-2	26437	0.193%	-4.939	7.85E-7	64472	0.151%	-0.675	0.5000	-3.231	1.23E-3
rs147770992	22:46795742	T/C	*CELSR1*	FEV1	never	EA	-764.555	4.17E-5	-0.809	3.30E-2	24497	0.024%	-4.419	9.93E-6	2940	0.011%	0.329	0.7421	-3.931	8.45E-5

Supplementary Table 3: Association results for SNPs identified in single variant association discovery analyses ($P<10^{-4}$), located in known lung function regions. Variants that are in regions previously identified as showing association with lung function traits. For each variant, the result presented is for the trait, smoking and ancestry combination for which the strongest association was identified.

							Discovery Analysis				Discovery meta-analysis				
							SpiroMeta Consortium		CHARGE Consortium						
rs id		Effect / other allele	(Nearest) Gene	Trait	Smoking subset (ever /never smokers / all)	Ancestry subset (both [EA+AA] / EA only)	Beta	P-value	Beta	P-value	N	Effect Allele Frequency (EAF)	Z-score	P-value	Previous Associations
rs1192415	1:92077097	G/A	-	FEV1/FVC	all	both	-0.044	2.99E-4	-0.204	2.34E-3	68099	18.1%	-4.497	6.91E-6	rs1192404 (r2=0.951) - Associated with FEV1/FVC[39]
rs2571445	2:218683154	A/G	*TNS1*	FEV1	all	both	-0.032	1.16E-3	-16.757	2.11E-6	68116	36.8%	-5.631	1.79E-8	SNP associated with FEV1 [48]
rs12477314	2:239877148	T/C	-	FEV1/FVC	all	both	0.034	3.77E-3	0.318	2.42E-6	68099	18.6%	5.411	6.26E-8	SNP associated with FEV1/FVC [48]
rs7671167	4:89883979	C/T	*FAM13A*	FEV1/FVC	all	both	0.043	5.87E-6	0.307	3.60E-9	68099	48.8%	7.293	3.03E-13	COPD locus; rs2045517 (r2=0.664) - associated with FEV1/FVC [48,49]
rs2280099	4:90035549	G/A	*TIGD2*	FEV1/FVC	all	EA	-0.059	1.63E-6	-0.183	0.012	60381	17.8%	-4.870	1.12E-6	rs7671167 (r2=0.150), Cho FAM13A and rs2045517 (r2=0.192) - associated with FEV1/FVC [48]
rs10516526	4:106688904	G/A	*GSTCD*	FEV1	all	EA	0.076	9.41E-5	44.087	3.79E-9	60395	6.4%	6.943	3.83E-12	SNP previously associated with FEV1[50]
rs13118928	4:145486389	G/A	-	FEV1/FVC	all	both	0.034	4.08E-4	0.407	7.41E-14	68099	37.9%	7.975	1.53E-15	rs138641402 (r2>0.9) - associated with FEV1/FVC [48]
rs11168048	5:147842353	C/T	*HTR4*	FEV1/FVC	all	both	0.045	3.92E-6	0.405	3.51E-14	68099	39.3%	8.673	4.19E-18	rs1985524 (r2=1) associated with FEV1 [48]
rs1422795	5:156936364	C/T	*ADAM19*	FEV1/FVC	all	both	-0.037	2.35E-4	-0.362	1.76E-11	68099	36.7%	-7.457	8.86E-14	SNP associated with FEV1/FVC [48]
rs2070600	6:32151443	T/C	*AGER*	FEV1/FVC	all	both	0.049	2.56E-2	0.900	2.33E-11	68099	4.6%	6.593	4.31E-11	SNP associated with FEV1/FVC [48]
rs17280293	6:142688969	G/A	*GPR126*	FEV1/FVC	all	both	0.125	3.29E-5	0.962	6.72E-9	68099	2.4%	6.993	2.69E-12	rs148274477 (r2=0.85) associated with FEV1/FVC [51]
rs11155242	6:142691549	C/A	*GPR126*	FEV1/FVC	all	EA	0.040	6.66E-4	0.503	1.17E-12	60381	19.2%	7.545	4.51E-14	rs262129 (r2=0.522) - associated with FEV1/FVC [48]
rs16909898	9:98231008	G/A	*LOC100507346*	FEV1/FVC	all	EA	-0.041	9.99E-3	-0.448	1.19E-6	60381	9.9%	-5.309	1.10E-7	SNP associated with FEV1/FVC [48]
rs12684650	9:139110654	T/C	*QSOX2*	FVC	all	both	-0.032	2.00E-3	-16.905	1.22E-4	68115	28.1%	-4.866	1.14E-6	rs10858246 (r2=0.887) - associated with FVC [51]
rs7068966	10:12277992	T/C	*CDC123*	FEV1/FVC	all	both	0.027	4.79E-3	0.295	1.77E-8	68099	48.9%	6.097	1.08E-9	rs7090277(r2=1) - associated with FEV1/FVC[39]
rs241	10:78444456	G/T	-	FEV1	never	both	0.029	0.027	19.154	4.15E-5	28519	50.0%	4.517	6.26E-6	rs2637254 (r2=0.657) - associated with FEV1 [48]
rs11555762	11:43876698	T/C	*HSD17B12*	FVC	all	EA	-0.036	5.89E-4	-16.963	1.80E-4	60394	30.3%	-5.015	5.30E-7	rs4237643 (r2=0.594) - associated with FVC [52]
rs10843206	12:28722756	C/T	-	FVC	all	EA	0.041	2.09E-5	18.870	6.80E-6	60394	50.5%	6.103	1.04E-9	rs2348418 (r2=0.853)- associated with FVC [51]
rs11172113	12:57527283	C/T	*LRP1*	FEV1/FVC	never	both	0.033	1.32E-2	0.305	2.61E-5	28504	40.7%	4.776	1.79E-6	SNP associated with FEV1/FVC [48]
rs2173958	12:115179246	G/T	*TBX3*	FEV1	all	both	-0.031	1.58E-3	-12.325	2.72E-4	68116	42.5%	-4.705	2.53E-6	rs10850377 (r2=0.273)- associated with FEV1 [51]
rs10498635	14:93103309	T/C	*RIN3*	FEV1	all	both	0.037	2.93E-3	20.912	3.20E-6	68116	17.0%	5.407	6.42E-8	rs117068593 (r2=0.991) - associated with FEV1[48]

rs id		Effect / other allele	(Nearest) Gene	Trait	Smoking subset (ever /never smokers / all)	Ancestry subset (both [EA+AA] / EA only)	Discovery Analysis				Discovery meta-analysis				Previous Associations
							SpiroMeta Consortium		CHARGE Consortium						
							Beta	P-value	Beta	P-value	N	Effect Allele Frequency (EAF)	Z-score	P-value	
rs12899618	15:71645120	A/G	*THSD4*	FEV1/FVC	all	EA	-0.063	1.85E-6	-0.420	4.90E-8	60381	15.3%	-7.109	1.17E-12	rs10851839 (r2=0.349) associated with FEV1/FVC [50]
rs8034191	15:78806023	C/T	*AGPHD1*	FEV1	ever	EA	-0.064	6.64E-6	-29.287	1.19E-6	27269	34.1%	-6.559	5.40E-11	Smoking/COPD locus [53]
rs12447804	16:58075282	T/C	*MMP15*	FEV1/FVC	all	both	-0.019	0.105	-0.300	6.94E-6	68099	19.3%	-4.502	6.72E-6	SNP associated with FEV1/FVC [48]
rs34093919	19:41117300	A/G	*LTBP4*	FEV1/FVC	all	EA	0.191	2.24E-5	0.656	5.57E-3	60381	1.3%	4.723	2.33E-6	rs113473882 (r2=0.878), associated with FEV1/FVC.[51]

Supplementary Table 4: Single variant association result for the seven novel signals, in smoking and ancestry subgroups. Smoking subgroups are ever smokers, never smokers and all individuals. Ancestry subgroups are European Ancestry only (EA) and both ancestries combined (European and African ancestries).

SNP	Trait	Ancestry	Smoking Analysis	CHARGE Beta	CHARGE P-value	SpiroMeta Beta	SpiroMeta P-value	Meta Z-score	Meta P-value
rs2322659	FVC	EA	ever	22.632	4.67E-3	0.036	0.029	3.550	3.85E-4
			never	33.311	2.53E-5	0.028	0.089	4.088	4.36E-5
			all	27.341	2.82E-7	0.032	7.60E-3	5.597	**2.18E-8**
		both	ever	14.976	0.031	0.036	0.029	3.027	2.47E-3
			never	25.048	1.66E-4	0.028	0.089	3.889	1.01E-4
			all	21.781	2.94E-6	0.032	7.60E-3	5.278	1.30E-7
rs1448044	FVC	EA	ever	19.713	3.47E-3	0.056	8.48E-5	4.763	1.90E-6
			never	6.620	0.323	-0.008	0.591	0.332	0.740
			all	10.966	0.014	0.024	0.022	3.317	9.11E-4
		both	ever	18.628	2.02E-3	0.056	8.48E-5	4.813	**1.49E-6**
			never	-0.395	0.946	-0.008	0.591	-0.392	0.695
			all	6.963	0.083	0.024	0.022	2.712	6.68E-3
rs1294421	FEV1/FVC	EA	ever	-0.174	0.064	-0.045	9.19E-4	-3.524	4.25E-4
			never	-0.204	0.013	-0.029	0.028	-3.284	1.02E-3
			all	-0.203	3.64E-4	-0.037	1.22E-4	-5.089	3.60E-7
		both	ever	-0.197	0.026	-0.045	9.19E-4	-3.741	1.83E-4
			never	-0.230	2.11E-3	-0.029	0.028	-3.734	1.88E-4
			all	-0.222	3.83E-5	-0.037	1.22E-4	-5.479	**4.27E-8**
rs3849969	FEV1	EA	ever	18.212	5.40E-3	0.035	0.021	3.584	3.39E-4
			never	13.253	0.030	0.038	1.01E-2	3.336	8.49E-4
			all	14.486	5.41E-4	0.036	6.99E-4	4.749	2.04E-6
		both	ever	14.775	9.70E-3	0.035	0.021	3.426	6.12E-4
			never	13.527	9.38E-3	0.038	0.010	3.609	3.07E-4
			all	13.095	3.83E-4	0.036	6.99E-4	4.767	**1.87E-6**
rs1200345	FEV1/FVC	EA	ever	-0.340	2.05E-4	-0.031	0.020	-4.279	1.87E-5
			never	-0.213	7.17E-3	-0.018	0.171	-2.853	4.33E-3
			all	-0.216	9.02E-5	-0.025	9.92E-3	-4.586	**4.51E-6**
		both	ever	-0.263	2.00E-3	-0.031	0.020	-3.812	1.38E-4
			never	-0.216	2.62E-3	-0.018	0.171	-3.158	1.59E-3
			all	-0.190	2.45E-4	-0.025	9.92E-3	-4.396	1.10E-5
rs1859962	FEV1	EA	ever	15.508	6.18E-3	0.040	2.99E-3	3.974	7.06E-5
			never	15.789	3.22E-3	0.012	0.370	2.736	6.22E-3
			all	15.389	2.75E-5	0.026	7.50E-3	4.876	**1.08E-6**
		both	ever	14.594	4.71E-3	0.040	2.99E-3	4.012	6.03E-5
			never	11.613	0.016	0.012	0.370	2.403	0.016
			all	13.308	7.43E-5	0.026	7.50E-3	4.681	2.85E-6
rs6088813	FVC	EA	ever	-7.325	0.261	-0.017	0.232	-1.627	0.104
			never	-10.242	0.111	-0.029	0.036	-2.559	0.011
			all	-13.728	1.35E-3	-0.023	0.023	-3.882	1.04E-4
		both	ever	-10.791	0.067	-0.017	0.232	-2.167	0.030
			never	-14.142	0.013	-0.029	0.036	-3.207	1.34E-3
			all	-16.157	3.44E-5	-0.023	0.023	-4.634	**3.58E-6**

Supplementary Table 5: Single variant association result for rs1448044 and FVC in ever smokers and never smokers separately, and in all samples combined. Replication Result presented includes data from UK Biobank and UKHLS only (result from NEO available only from ever smokers). Original combined discovery and replication result in ever smokers, including NEO: P= $2.22x10^{-11}$.

				Consortium level Discovery Analysis									
				CHARGE Consortium		SpiroMeta Consortium		Discovery Meta-analysis		Overall Replication		Combined Discovery + Replication Result	
SNP	Trait	Ancestry	Smoking Analysis	Beta	P-value	Beta	P-value	Z-score	P-value	Z-score	P-value	Z-score	P-value
rs1448044	FVC	both	ever	18.628	2.02E-3	0.056	8.48E-5	4.813	1.49E-6	4.163	3.14E-5	6.354	2.10E-10
			never	-0.395	0.946	-0.008	0.591	-0.392	0.695	3.809	1.40E-4	2.385	1.71E-2
			all	6.963	0.083	0.024	0.022	2.712	6.68E-3	5.638	1.72E-8	5.638	4.22E-9

Supplementary Table 6: Association results for all genes identified in discovery SKAT analyses (meta-analysis P<10^{-4}). N SNPs is the number of SNPs included in the SKAT test. For the two each genes with evidence of replication, a conditional analysis was carried out, conditioning on the most significantly associated individual SNP within that gene. In each case, the individual SNP id is given, along with conditional SKAT P-value when that SNP has been conditioned on. Only the trait, smoking ancestry combination for which each gene was most significantly associated is shown.

					Discovery Analysis							
					CHARGE Consortium		SpiroMeta Consortium		Meta-Analysis	Replication Analysis		
GENE	Trait	Smoking subset (ever /never smokers / all)	Ancestry subset (both [EA+AA] / EA only)	SNP set	N SNPs	P-value	N SNPs	P-value	P-value	N SNPs	P-value	Notes
GPR126	FEV1/FVC	all	both	exonic	25	9.98E-10	20	4.42E-5	1.40E-12	11	2.03E-40	Driven by rs17280293 (known signal); conditional P=0.287
LTBP4	FEV1/FVC	all	EA	exonic	25	3.87E-3	25	3.49E-5	2.27E-6	17	2.23E-15	Driven by rs34093919 (known signal); conditional P=0.4552
NCF4	FEV1/FVC	ever	EA	exonic	12	8.08E-6	11	4.05E-2	5.21E-6	3	0.805	
LY6G6D	FEV1/FVC	ever	both	exonic	3	6.60E-5	2	8.83E-3	8.96E-6	NA	NA	
IL17RB	FEV1/FVC	all	EA	LOF	3	1.91E-3	2	5.96E-4	1.67E-5	NA	NA	
CPVL	FEV1	never	EA	LOF	3	6.79E-5	3	2.90E-2	2.78E-5	NA	NA	
SEPT12	FVC	never	both	exonic	12	1.59E-4	8	1.54E-2	3.41E-5	3	0.525	
GUCA1C	FVC	ever	both	exonic	4	3.77E-4	3	7.89E-3	4.08E-5	1	0.087	
CC2D2B	FEV1	all	both	exonic	9	2.64E-4	6	1.18E-2	4.27E-5	2	0.800	
ALOXE3	FVC	never	EA	exonic	15	1.81E-2	13	1.87E-4	4.60E-5	4	0.599	
ZNF491	FEV1/FVC	never	both	LOF	2	9.89E-5	2	4.25E-2	5.63E-5	NA	NA	
FAS	FVC	never	both	exonic	6	1.36E-3	3	4.12E-3	7.34E-5	2	0.026	
USP49	FEV1	all	both	exonic	8	5.77E-4	7	1.15E-2	8.56E-5	2	0.776	
ANO6	FEV1/FVC	ever	both	exonic	20	1.46E-2	16	4.73E-4	8.91E-5	6	0.495	
RAD18	FEV1/FVC	all	both	exonic	13	3.06E-3	12	2.47E-3	9.66E-5	2	0.776	
MXI1	FVC	never	both	exonic	3	1.35E-3	2	5.75E-3	9.93E-5	4	0.726	

Supplementary Table 7: Association results for all genes identified in discovery Weighted sum test (WST) test analyses (P<10^{-4}). N SNPs is the number of SNPs included in the WST. Betas values for SpiroMeta Consortium and Replication results reflect effect-size estimates on an inverse-normal transformed scale. For the CHARGE Consortium, beta values represent untransformed trait effect estimates. Only the trait, smoking ancestry combination for which each gene was most significantly associated is shown.

					Discovery Analysis							Replication Analysis		
					CHARGE Consortium			SpiroMeta Consortium			Meta-Analysis			
GENE	Trait	Smoking subset (ever /never smokers / all)	Ancestry subset (both [EA+AA] / EA only)	SNP set	N SNPs	Beta	P-value	N SNPs	Beta	P-value	P-value	N SNPs	Beta	P-value
EDARADD	FEV1/FVC	all	EA	exonic	4	-0.1729	2.77E-5	2	-0.0067	3.21E-2	3.93E-6	1	0.0028	0.2150
AAMP	FVC	all	both	exonic	9	-5.1496	6.35E-3	5	-0.0142	6.29E-4	2.48E-5	3	0.0006	0.6604
LY6G6D	FEV1	all	EA	exonic	2	-13.4532	3.56E-4	2	-0.0108	2.44E-2	2.72E-5	NA	NA	NA
GSTM5	FEV1	never	EA	exonic	3	-13.9157	3.64E-3	4	-0.0130	3.51E-3	3.79E-5	2	0.0022	0.3585
RCN3	FVC	all	EA	exonic	9	6.1012	2.39E-3	8	0.0108	5.58E-3	4.09E-5	5	0.0013	0.2100
TMPRSS7	FEV1	never	both	LOF	3	-14.4113	7.11E-4	2	-0.1528	2.19E-2	4.86E-5	NA	NA	NA
GATA5	FEV1/FVC	ever	EA	exonic	3	-0.2505	1.74E-3	3	-0.0099	1.02E-2	5.16E-5	3	-0.0011	0.5227
CHTF18	FEV1	never	both	exonic	49	-3.7700	4.43E-4	42	-0.0038	3.20E-2	5.19E-5	15	0.0008	0.3193
SALL2	FEV1/FVC	all	both	exonic	13	-0.0708	2.26E-3	10	-0.0059	7.88E-3	5.48E-5	NA	NA	NA
SH2D3A	FVC	all	EA	LOF	2	13.5166	1.25E-3	2	0.0817	1.56E-2	5.52E-5	NA	NA	NA
NUP188	FEV1	ever	both	exonic	36	3.7176	1.93E-3	24	0.0032	1.52E-2	8.24E-5	10	-0.0005	0.5733
RBPMS2	FEV1/FVC	all	both	exonic	2	-0.1670	2.06E-3	2	-0.0108	1.51E-2	8.72E-5	2	-0.0029	0.0657

Supplementary Table 8: Evidence for the role of novel variants identified in single variant association analyses as eQTLs For each probeset variants were ranked firstly according to their correlation with the sentinel SNP (r2) and secondly by eQTL P value, and only the top ranked SNP for each probeset is presented (eQTL SNP). Results in bold indicate there is co-localisation of the GWAS and eQTL signal, that is sentinel SNP and the top eSNP for that gene/tissue combination have r2>0.9. Lung† indicates result from the lung resection cohort, Blood‡ indicates the result is from the Westra et al.[42] blood dataset; all other results from GTEx. All identified eQTLs are cis.

sentinel	Gene	eQTL SNP	r2 with sentinel	tissue	P-value
rs1200345	*JMJD7-PLA2G4B*	rs1200349	1.000	Lung†	1.54E-6
rs1200345	*LTK*	rs11632399	1.000	Lung†	6.90E-65
		rs2297378	0.919	Small_Intestine_Terminal_Ileum	3.94E-6
		rs7178957	0.942	Artery_Tibial	1.40E-5
rs1200345	*MAPKBP1*	rs11632399	1.000	Lung†	3.66E-29
rs1200345	*NDUFAF1*	rs1200345	1.000	Muscle_Skeletal	7.71E-7
		rs28860492	0.996	Lung	1.00E-5
**rs1200345**	***RPAP1***	rs1200349	1.000	Blood‡	6.93E-21
		rs1200345	1.000	Esophagus_Mucosa	2.33E-17
		**rs1200349**	**1.000**	**Lung†**	**2.52E-16**
		**rs1200345**	**1.000**	**Nerve_Tibial**	**3.31E-15**
		rs1200345	1.000	Colon_Transverse	3.37E-13
		rs1200345	1.000	Artery_Aorta	2.18E-12
		**rs1200345**	**1.000**	**Adipose_Visceral_Omentum**	**4.94E-11**
		rs1200345	1.000	Thyroid	2.47E-10
		rs1200345	1.000	Whole_Blood	1.99E-9
		**rs1200345**	**1.000**	**Breast_Mammary_Tissue**	**2.43E-9**
		rs1200345	1.000	Adrenal_Gland	1.60E-8
		rs1200345	1.000	Esophagus_Gastroesophageal_Junction	5.55E-8
		rs1200345	1.000	Brain_Putamen_basal_ganglia	1.30E-7
		rs1200345	1.000	Testis	4.71E-7
		**rs1200345**	**1.000**	**Vagina**	**5.23E-7**
		rs2297380	0.838	Brain_Frontal_Cortex_BA9	8.06E-6
rs1200345	*SPTBN5*	rs11632399	1.000	Lung†	7.12E-8
		rs1200345	1.000	Adipose_Subcutaneous	1.09E-7
		rs1200345	1.000	Lung	2.82E-7
		rs1200345	1.000	Cells_Transformed_fibroblasts	2.07E-6
		rs1200345	1.000	Adipose_Visceral_Omentum	2.44E-6
		rs1200345	1.000	Thyroid	3.50E-6
		rs1200345	1.000	Muscle_Skeletal	1.48E-5
		rs1200345	1.000	Artery_Aorta	1.67E-5
		rs1200345	1.000	Artery_Tibial	1.73E-5
rs1200345	*TYRO3*	rs1200345	1.000	Lung†	1.50E-24
rs2322659	*AC093391.2*	rs1030765	0.807	Artery_Tibial	3.82E-6
rs2322659	*CCNT2*	rs2322659	1.000	Lung†	2.37E-6
rs2322659	*CXCR4*	rs309134	0.837	Blood‡	3.98E-6
rs2322659	*DARS*	rs2322659	1.000	Blood‡	1.06E-57

sentinel	Gene	eQTL SNP	r2 with sentinel	tissue	P-value
		rs2322659	1.000	Lung†	3.94E-26
		rs2322659	1.000	Thyroid	3.48E-8
		rs2304599	0.903	Artery_Tibial	2.46E-5
rs2322659	*MCM6*	rs2322659	1.000	Blood‡	1.91E-119
		rs2322659	1.000	Lung†	3.29E-10
		rs2322659	1.000	Esophagus_Muscularis	3.40E-7
		rs2322659	1.000	Thyroid	7.90E-7
		rs2322659	1.000	Nerve_Tibial	1.46E-6
		rs2322659	1.000	Artery_Aorta	2.55E-6
rs2322659	*UBXN4*	rs2322659	1.000	Skin_Sun_Exposed_Lower_leg	4.19E-8
		rs1030764	0.908	Lung†	2.60E-6
rs2322659	*ZRANB3*	rs1470457	0.946	Lung†	4.36E-6
**rs3849969**	***AGAP5***	**rs10740417**	**1.000**	**Testis**	**1.24E-6**
		rs10740417	1.000	Muscle_Skeletal	1.87E-5
rs3849969	*CAMK2G*	rs3812637	1.000	Blood‡	1.67E-5
rs3849969	*FUT11*	rs11000765	1.000	Nerve_Tibial	1.48E-5
		rs10762556	0.995	Muscle_Skeletal	2.12E-5
		rs10762556	0.995	Cells_Transformed_fibroblasts	3.86E-5
**rs3849969**	***NDST2***	**rs3812637**	**1.000**	**Blood‡**	**5.36E-47**
rs3849969	*PLAU,C10orf55*	rs3812637	1.000	Blood‡	1.57E-8
rs3849969	*SEC24C*	rs3849969	1.000	Stomach	9.57E-7
rs6088813	*CEP250*	rs4911494	1.000	Blood‡	4.14E-8
rs6088813	*EDEM2*	rs6088813	1.000	Cells_Transformed_fibroblasts	7.06E-6
		rs1540927	0.996	Blood‡	3.05E-5
rs6088813	*EIF6*	rs6141548	1.000	Blood‡	3.96E-31
		rs725908	0.996	Cells_Transformed_fibroblasts	3.12E-5
**rs6088813**	***ERGIC3***	**rs4911496**	**1.000**	**Brain_Hippocampus**	**1.20E-6**
rs6088813	*FAM83C*	rs4911494	1.000	Skin_Sun_Exposed_Lower_leg	1.82E-6
rs6088813	*GDF5*	rs6142364	1.000	Lung	6.26E-9
		rs4353719	1.000	Lung†	2.12E-7
		rs6142360	0.996	Esophagus_Muscularis	2.95E-5
rs6088813	*GGT7*	rs6088817	1.000	Lung†	1.32E-5
rs6088813	*RP3-477O4.16*	rs6142349	1.000	Testis	1.16E-7
		rs4911494	1.000	Skin_Sun_Exposed_Lower_leg	2.24E-6
		rs6142364	1.000	Nerve_Tibial	4.63E-5
rs6088813	*RPL36P4*	rs6088813	1.000	Cells_Transformed_fibroblasts	2.39E-10
		rs6088813	1.000	Adipose_Subcutaneous	6.89E-10
		rs6088813	1.000	Nerve_Tibial	1.05E-6
		rs1406948	0.937	Adrenal_Gland	1.20E-5
		rs6088813	1.000	Thyroid	1.40E-5
**rs6088813**	***UQCC1***	**rs6088809**	**1.000**	**Lung†**	**5.69E-24**
		**rs6088813**	**1.000**	**Blood‡**	**1.65E-11**
		**rs6142364**	**1.000**	**Cells_Transformed_fibroblasts**	**1.59E-28**

sentinel	Gene	eQTL SNP	r2 with sentinel	tissue	P-value
		rs6142364	1.000	Muscle_Skeletal	5.24E-11
		rs6142364	1.000	Lung	1.69E-10
		rs6088813	1.000	Nerve_Tibial	1.25E-8
		rs6088813	1.000	Stomach	7.92E-6
		rs6088813	1.000	Pancreas	1.31E-5

Supplementary Table 9: SIFT/Polyphen predictions for sentinel SNPs and proxies (r2>0.8). All sentinel SNPs and proxies (r2>0.8) were annotated using variant effect predictor and any SNP annotated as deleterious or damaging by either SIFT or PolyPhen identified.

**Sentinel**	**Proxy**	**R^2**	**Chr:Pos**	**Allele**	**Consequence**	**Gene**	**Transcript ID**	**Codons**	**SIFT consequence (score)**	**PolyPhen consequence (score)**
rs1200345	-	-	15:41527518	T	missense_variant	*RPAP1*	ENST00000304330	Gag/Aag	deleterious(0)	possibly_damaging(0.526)
rs1200345	-	-	15:41527518	T	missense_variant, NMD_transcript_variant	*RPAP1*	ENST00000562303	Gag/Aag	deleterious(0.01)	possibly_damaging(0.718)
rs1200345	-	-	15:41527518	T	missense_variant	*RPAP1*	ENST00000304330	Gag/Aag	deleterious(0)	possibly_damaging(0.526)
rs1200345	-	-	15:41527518	T	missense_variant	*RPAP1*	ENST00000561603	Gag/Aag	tolerated(0.05)	probably_damaging(0.996)
rs1200345	-	-	15:41527518	T	missense_variant, NMD_transcript_variant	*RPAP1*	ENST00000562303	Gag/Aag	deleterious(0.01)	possibly_damaging(0.718)
rs6088813	rs224331	0.895	20:35434589	A	missense_variant	*GDF5*	ENST00000374369	Gcc/Tcc	tolerated(0.8)	possibly_damaging(0.455)
rs6088813	rs224331	0.895	20:35434589	A	missense_variant	*GDF5*	ENST00000374372	Gcc/Tcc	tolerated(0.8)	possibly_damaging(0.455)
rs6088813	rs224331	0.895	20:35434589	A	missense_variant	*GDF5OS*	ENST00000374375	gCg/gAg	tolerated_low_confidence(0.59)	possibly_damaging(0.773)
rs6088813	rs224331	0.895	20:35434589	T	missense_variant	*GDF5OS*	ENST00000374375	gCg/gTg	tolerated_low_confidence(0.1)	possibly_damaging(0.691)

Supplementary Table 10: Protein and RNA expression results all implicated genes from the single variant association analyses. Implicated genes were those located at, or close to the position of the sentinel SNP, or were implicated through eQTL data (See Supplementary Table 10). Protein expression are qualitative antibody based protein profiles in the respiratory system for epithelial cells, pneumocytes and macrophages from the Human Protein Atlas. RNA expression is quantitative data estimating the transcript abundance of each protein-coding gene by RNA-seq from the Human Protein Atlas and GTEx. FPKM: Fragmments Per Kilobase gene model and Million reads. RPKM: Reads Per Kilobase gene model and Million mapped reads.

	Protein Expression				RNA Profile			
	Human Protein Atlas				Human Protein Atlas		GTEx	
**Gene**	**nasopharynx**	**bronchus**	**lung macrophages**	**lung pneumocytes**	**FKPM**	**Category**	**RPKM**	**Category**
LCT	low	medium	medium	not detected	0.0	not detected	0	not detected
FGF10	medium	medium	low	low	3.0	low	1.4	low
LY86	not detected	not detected	high	not detected	18.6	medium	7.4	low
SEC24C	medium	medium	medium	medium	24.4	medium	30.7	medium
RPAP1	high	high	medium	high	4.4	low	4.5	low
CASC17	NA	NA	NA	NA	NA	NA	NA	NA
UQCC1	medium	high	medium	medium	11.5	medium	6.4	low
NDST2	not detected	not detected	not detected	not detected	12.1	medium	0.8	low
PLAU	low	NA	low	low	15.1	medium	14	medium
C10orf55	not detected	low	not detected	not detected	0.1	not detected	0	not detected
CAMK2G	low	medium	not detected	not detected	16.8	medium	10.2	medium
FUT11	medium	medium	medium	high	7.1	low	5.5	low
AGAP5	medium	medium	not detected	low	1.1	low	2.2	low
SPTBN5	NA	NA	NA	NA	0.3	not detected	1	low
LTK	medium	medium	medium	low	9.5	low	13.6	medium
NDUFAF1	medium	medium	medium	medium	10.4	medium	6	low
MCM6	medium	medium	not detected	medium	5.4	low	7.2	low
DARS	high	medium	medium	medium	29.1	medium	17.8	medium
CXCR4	NA	NA	NA	NA	23.1	medium	59.2	high
AC093391.2	NA	NA	NA	NA	NA	NA	NA	NA
UBXN4	high	high	high	medium	27.8	medium	29.5	medium
CEP250	NA	NA	NA	NA	5.9	low	3.3	low
EIF6	medium	NA	medium	low	45.5	medium	39.3	medium
EDEM2	medium	medium	medium	medium	20.2	medium	11.9	medium
RPL36P4	NA	NA	NA	NA	NA	NA	NA	NA
ERGIC3	medium	medium	medium	low	87.5	high	52.2	high
GDF5	NA	NA	NA	NA	0.7	low	1.1	low
RP3-477O4.16	NA	NA	NA	NA	NA	NA	NA	NA
FAM83C	NA	NA	NA	NA	NA	NA	NA	NA
JMJD7-PLA2G4B	low	high	medium	not detected	3.8	low	NA	NA
MAPKBP1	medium	medium	low	not detected	3.8	low	4.9	low
TYRO3	medium	medium	high	not detected	2.4	low	4.1	low
CCNT2	high	high	high	high	10.3	medium	8.9	low
ZRANB3	medium	medium	medium	medium	0.6	low	0.3	not detected

GGT7	high	medium	medium	not detected	7.8	low	10.9	medium

Supplementary Table 11: Look-up of association results for SNPs at 7 of the 12 loci which showed allele frequency differences between individuals from different regions in the UK. Shown are the P-values for associations with the three lung function traits (FEV1, FVC and FEV1/FVC), in both the analyses restricted to EA individuals, or in the analysis of EA and AA individuals combined. The 7 loci shown are those with an available proxy (r^2>0.2) for the SNPs reported in [54].

					Discovery analysis P-values					
Discovery Analysis SNP			Population structure SNP		EA + AA			EA only		
SNP	CHR	POS	SNP	r^2 with analysed SNP	FEV1	FVC	FEV1/FVC	FEV1	FVC	FEV1/FVC
rs3754689	2	136590746	rs1042712	0.9093	0.066	1.95E-03	0.216	0.011	3.19E-04	0.433
rs4331786	4	38769408	rs7696175	0.3641	0.250	0.168	0.457	0.254	0.169	0.415
rs9378805	6	417727	rs9378805	1	0.250	0.130	0.765	0.191	0.109	0.913
rs3873379	6	31262169	rs3873375	1	0.406	0.242	0.786	0.464	0.360	0.799
rs755383	9	863635	rs11790408	0.2032	0.960	0.538	0.169	0.764	0.414	0.196
rs12785878	11	71167449	rs12797951	0.9259	0.484	0.686	0.471	0.526	0.825	0.404
rs61976859	14	31583512	rs17449560	0.2756	0.753	0.337	0.433	0.680	0.343	0.530

Supplementary Table 12: All traits results for the seven novel lung function loci. Two-sided P-values are given for the discovery stage meta-analysis consisting of up to 68,470 individuals from the SpiroMeta and CHARGE Consortia.

SNP	Chr:Pos	(Nearest) Gene(s)	Smoking	Ancestry	Effect / other allele	Effect Allele Frequency (Discovery)	FEV1		FVC		FEV1/FVC	
							meta.Z	meta.P	meta.Z	meta.P	meta.Z	meta.P
rs2322659	2:136555659	*LCT* (nonsynonymous)	All Individuals	EA Only	T/C	23.50%	4.5938	4.35E-06	5.5968	2.18E-08	-0.5893	0.556
rs1448044	5:44296986	*FGF10* (dist=8111), *NNT* (dist=591,318)	Ever Smokers	EA+AA	A/G	35.60%	2.9427	3.25E-03	4.8129	1.49E-06	-1.4533	0.146
rs1294421	6:6743149	*LY86* (dist=87933), *RREB1* (dist=364,681)	All Individuals	EA+AA	T/G	36.80%	-1.4521	0.146	1.6555	0.098	-5.4795	4.27E-08
rs3849969	10: 75525999	*SEC24C* (intronic)	All Individuals	EA+AA	T/C	29.40%	4.7665	1.87E-06	3.5369	4.05E-04	3.0351	2.40E-03
rs1200345	15: 41819716	*RPAP1* (nonsynonymous)	All Individuals	EA only	C/T	48.80%	-0.9685	0.333	1.3016	0.193	-4.5865	4.508E-06
rs1859962	17: 69108753	*CASC17* (intronic)	All Individuals	EA only	G/T	48.20%	4.8760	1.08E-06	3.4002	6.73E-04	3.2432	1.18E-03
rs6088813	20: 33975181	*UQCC1* (intronic)	All Individuals	EA+AA	C/A	36.70%	-3.5529	3.81E-04	-4.6343	3.582E-06	1.3266	0.185

Supplementary Table 13: Genomic Inflation Factors: consortium and meta-analysis level. The SpiroMeta analysis included EA individuals only, thus the λ values for EA only and EA+AA are identical.

**Trait**	**Smoking**	**Ancestry**	**SpiroMeta genomic inflation factor (λ)**	**CHARGE λ**	**Meta-analysis λ**
FEV_1	All Individuals	EA only	1.060	1.178	1.038
FEV_1	All Individuals	EA+AA	1.060	1.169	1.048
FEV_1	Ever Smokers	EA only	1.023	1.054	1.028
FEV_1	Ever Smokers	EA+AA	1.023	1.061	1.027
FEV_1	Never Smokers	EA only	1.020	1.040	1.018
FEV_1	Never Smokers	EA+AA	1.020	1.053	1.033
FVC	All Individuals	EA only	1.072	1.146	1.037
FVC	All Individuals	EA+AA	1.072	1.142	1.039
FVC	Ever Smokers	EA only	1.001	1.066	1.008
FVC	Ever Smokers	EA+AA	1.001	1.070	1.014
FVC	Never Smokers	EA only	1.031	1.029	1.046
FVC	Never Smokers	EA+AA	1.031	1.028	1.040
FEV_1/FVC	All Individuals	EA only	1.037	1.135	1.039
FEV_1/FVC	All Individuals	EA+AA	1.037	1.136	1.042
FEV_1/FVC	Ever Smokers	EA only	1.018	1.094	1.031
FEV_1/FVC	Ever Smokers	EA+AA	1.018	1.104	1.034
FEV_1/FVC	Never Smokers	EA only	1.006	1.036	1.022
FEV_1/FVC	Never Smokers	EA+AA	1.006	1.016	1.017

References

1. The Atherosclerosis Risk in Communities (ARIC) Study: design and objectives. The ARIC investigators. *Am J Epidemiol* 1989; **129**(4): 687-702.

2. Strachan DP, Rudnicka AR, Power C, et al. Lifecourse influences on health among British adults: effects of region of residence in childhood and adulthood. *Int J Epidemiol* 2007; **36**(3): 522-31.

3. Marossy AE, Strachan DP, Rudnicka AR, Anderson HR. Childhood chest illness and the rate of decline of adult lung function between ages 35 and 45 years. *Am J Respir Crit Care Med* 2007; **175**(4): 355-9.

4. Fried LP, Borhani NO, Enright P, et al. The cardiovascular health study: design and rationale. *Ann Epidemiol* 1991; **1**(3): 263-76.

5. Enright PL, Kronmal RA, Higgins M, Schenker M, Haponik EF. Spirometry reference values for women and men 65 to 85 years of age. *Am Rev Respir Dis* 1993; **147**(125): e133.

6. Zemunik T, Boban M, Lauc G, et al. Genome-wide association study of biochemical traits in Korcula Island, Croatia. *Croatian medical journal* 2009; **50**(1): 23-33.

7. Higgins M, Province M, Heiss G, et al. NHLBI Family Heart Study: objectives and design. *Am J Epidemiol* 1996; **143**(12): 1219-28.

8. Smith BH, Campbell A, Linksted P, et al. Cohort Profile: Generation Scotland: Scottish Family Health Study (GS:SFHS). The study, its participants and their potential for genetic research on health and illness. *Int J Epidemiol* 2013; **42**(3): 689-700.

9. Waterer GW, Wan JY, Kritchevsky SB, et al. Airflow Limitation is Underrecognized in Well-Functioning Older People. *J Am Geriatr Soc* 2001; **49**(8): 1032-8.

10. Thuesen BH, Cerqueira C, Aadahl M, et al. Cohort Profile: the Health2006 cohort, research centre for prevention and health. *Int J Epidemiol* 2014; **43**(2): 568-75.

11. Jorgensen T, Borch-Johnsen K, Thomsen TF, Ibsen H, Glumer C, Pisinger C. A randomized non-pharmacological intervention study for prevention of ischaemic heart disease: baseline results Inter99. *Eur J Cardiovasc Prev Rehabil* 2003; **10**(5): 377-86.

12. Taylor Jr HA, Wilson JG, Jones DW, et al. Toward resolution of cardiovascular health disparities in African Americans: design and methods of the Jackson Heart Study. *Ethn Dis* 2005; **15**(4 Suppl 6): S6-4.

13. American Thoracic Society. Standardization of spirometry: 1994 update. *Am J Respir Crit Care Med* 1995; **152**: 1107-36.

14. Holle R, Happich M, Lowel H, Wichmann HE, Group MKS. KORA--a research platform for population based health research. *Gesundheitswesen* 2005; **67 Suppl 1**: S19-25.

15. Wichmann HE, Gieger C, Illig T, Group MKS. KORA-gen--resource for population genetics, controls and a broad spectrum of disease phenotypes. *Gesundheitswesen* 2005; **67 Suppl 1**: S26-30.

16. Miller MR, Hankinson J, Brusasco V, et al. Standardisation of spirometry. *Eur Respir J* 2005; **26**(2): 319-38.

17. Peters A, Doring A, Ladwig KH, et al. [Multimorbidity and successful aging: the population-based KORA-Age study]. *Zeitschrift fur Gerontologie und Geriatrie* 2011; **44 Suppl 2**: 41-54.

18. Deary IJ, Gow AJ, Taylor MD, et al. The Lothian Birth Cohort 1936: a study to examine influences on cognitive ageing from age 11 to age 70 and beyond. *BMC Geriatr* 2007; **7**: 28.

19. Bild DE, Bluemke DA, Burke GL, et al. Multi-Ethnic Study of Atherosclerosis: objectives and design. *Am J Epidemiol* 2002; **156**(9): 871-81.

20. Kaufman JD, Adar SD, Allen RW, et al. Prospective study of particulate air pollution exposures, subclinical atherosclerosis, and clinical cardiovascular disease: The Multi-Ethnic Study of Atherosclerosis and Air Pollution (MESA Air). *Am J Epidemiol* 2012; **176**(9): 825-37.

21. Pellegrino R, Viegi G, Brusasco V, et al. Interpretative strategies for lung function tests. *Eur Respir J* 2005; **26**(5): 948-68.

22. Hankinson JL, Kawut SM, Shahar E, Smith LJ, Stukovsky KH, Barr RG. Performance of American Thoracic Society-recommended spirometry reference values in a multiethnic sample of adults: the multi-ethnic study of atherosclerosis (MESA) lung study. *CHEST Journal* 2010; **137**(1): 138-45.

23. Rodriguez J, Jiang R, Johnson WC, MacKenzie BA, Smith LJ, Barr RG. The association of pipe and cigar use with cotinine levels, lung function, and airflow obstruction: a cross-sectional study. *Ann Intern Med* 2010; **152**(4): 201-10.

24. Lind L, Fors N, Hall J, Marttala K, Stenborg A. A comparison of three different methods to evaluate endothelium-dependent vasodilation in the elderly: the Prospective Investigation of the Vasculature in Uppsala Seniors (PIVUS) study. *Arteriosclerosis, thrombosis, and vascular biology* 2005; **25**(11): 2368-75.

25. Hofman A, Brusselle GG, Murad SD, et al. The Rotterdam Study: 2016 objectives and design update. *Eur J Epidemiol* 2015; **30**(8): 661-708.

26. Celli BR, MacNee W, ATS/ERS Task Force. Standards for the diagnosis and treatment of patients with COPD: a summary of the ATS/ERS position paper. *Eur Respir J* 2004; **23**(6): 932-46.

27. Martin BW, Ackermann-Liebrich U, Leuenberger P, et al. SAPALDIA: methods and participation in the cross-sectional part of the Swiss Study on Air Pollution and Lung Diseases in Adults. *Sozial- und Praventivmedizin* 1997; **42**(2): 67-84.

28. Ackermann-Liebrich U, Kuna-Dibbert B, Probst-Hensch NM, et al. Follow-up of the Swiss Cohort Study on Air Pollution and Lung Diseases in Adults (SAPALDIA 2) 1991-2003: methods and characterization of participants. *Sozial- und Praventivmedizin* 2005; **50**(4): 245-63.

29. John U, Greiner B, Hensel E, et al. Study of Health In Pomerania (SHIP): a health examination survey in an east German region: objectives and design. *Soz Praventivmed* 2001; **46**(3): 186-94.

30. Volzke H, Alte D, Kohlmann T, Ludemann J, Nauck M, John U, Meng W. Reference intervals of serum thyroid function tests in a previously iodine-deficient area. *Thyroid* 2005; **15**(3): 279-85.

31. Nelson SB, Gardner RM, Crapo RO, Jensen RL. Performance evaluation of contemporary spirometers. *Chest* 1990; **97**(2): 288-97.

32. Standardization of spirometry--1987 update. Statement of the American Thoracic Society. *Am Rev Respir Dis* 1987; **136**(5): 1285-98.

33. Wain LV, Shrine N, Miller S, et al. Novel insights into the genetics of smoking behaviour, lung function, and chronic obstructive pulmonary disease (UK BiLEVE): a genetic association study in UK Biobank. *Lancet Respir Med* 2015; **3**(10): 769-81.

34. Raitakari OT, Juonala M, Ronnemaa T, et al. Cohort profile: the cardiovascular risk in Young Finns Study. *International journal of epidemiology* 2008; **37**(6): 1220-6.

35. Mahajan A, Robertson N, Rayner W. Exome-Chip Quality Control SOP. Version 5, 2012-11-20. 2012; .

36. Illumina Inc. Illumina GenCall Data Analysis Software. 2005; .

37. Goldstein JI, Crenshaw A, Carey J, et al. zCall: a rare variant caller for array-based genotyping: Genetics and population analysis. *Bioinformatics* 2012; **28**(19): 2543-5.

38. Grove ML, Yu B, Cochran BJ, et al. Best practices and joint calling of the HumanExome BeadChip: the CHARGE Consortium. *PLoS One* 2013; **8**(7): e68095.

39. Wain LV, Shrine N, Soler Artigas M, et al. Genome-wide association analyses for lung function and chronic obstructive pulmonary disease identify new loci and potential druggable targets. 2017; .

40. The 1000 Genomes Project Consortium. An integrated map of genetic variation from 1,092 human genomes. *Nature* 2012; **491**(7422): 56-65.

41. Huang J, Howie B, McCarthy S, et al. Improved imputation of low-frequency and rare variants using the UK10K haplotype reference panel. *Nat Commun* 2015; **6**: 8111.

42. Westra H, Peters MJ, Esko T, et al. Systematic identification of trans eQTLs as putative drivers of known disease associations. *Nat Genet* 2013; **45**(10): 1238-43.

43. Lonsdale J, Thomas J, Salvatore M, et al. The Genotype-Tissue Expression (GTEx) project. *Nat Genet* 2013; **45**(6): 580-5.

44. Obeidat M, Miller S, Probert K, et al. GSTCD and INTS12 Regulation and Expression in the Human Lung. *PLoS ONE* 2013; **8**(9): e74630.

45. Lamontagne M, Couture C, Postma DS, et al. Refining susceptibility loci of chronic obstructive pulmonary disease with lung eqtls. *PLoS One* 2013; **8**(7): e70220.

46. Hao K, Bossé Y, Nickle DC, et al. Lung eQTLs to help reveal the molecular underpinnings of asthma. *PLoS Genet* 2012; **8**(11): e1003029.

47. Irizarry RA, Hobbs B, Collin F, Beazer-Barclay YD, Antonellis KJ, Scherf U, Speed TP. Exploration, normalization, and summaries of high density oligonucleotide array probe level data. *Biostatistics* 2003; **4**(2): 249-64.

48. Soler Artigas M, Loth DW, Wain LV, et al. Genome-wide association and large-scale follow up identifies 16 new loci influencing lung function. *Nat Genet* 2011; **43**(11): 1082-90.

49. Cho MH, Boutaoui N, Klanderman BJ, et al. Variants in FAM13A are associated with chronic obstructive pulmonary disease. *Nat Genet* 2010; **42**(3): 200-2.

50. Repapi E, Sayers I, Wain LV, et al. Genome-wide association study identifies five loci associated with lung function. *Nat Genet* 2010; **42**(1): 36-44.

51. Soler Artigas M, Wain LV, Miller S, et al. Sixteen new lung function signals identified through 1000 Genomes Project reference panel imputation. *Nat Commun* 2015; **6**: 8658.

52. Loth DW, Soler Artigas M, Gharib SA, et al. Genome-wide association analysis identifies six new loci associated with forced vital capacity. *Nat Genet* 2014; **46**(7): 669-77.

53. Pillai SG, Ge D, Zhu G, et al. A Genome-Wide Association Study in Chronic Obstructive Pulmonary Disease (COPD): Identification of Two Major Susceptibility Loci. *PLoS Genet* 2009; **5**(3): e1000421.

54. Burton PR, Clayton DG, Cardon LR, et al. Genome-wide association study of 14,000 cases of seven common diseases and 3,000 shared controls. *Nature* 2007; **447**(7145): 661-78.
