# Supplementary material for: Meta-analysis of exome array data identifies six novel genetic loci for lung function
Source: Wellcome Open Res. 2018 Aug 7;3:4. Originally published 2018 Jan 12. [Version 3] doi: 10.12688/wellcomeopenres.12583.3 (PMC6081985; doi:10.12688/wellcomeopenres.12583.3)
Supplement: Peer review history

# Peer review: Referee response for version 2

**DOI:** 10.21956/wellcomeopenres.15514.r33384

**Published:** 2018-07-03

**Authors:** Rachel M. Freathy, Robin Beaumont

**Affiliations:** 1 Institute of Biomedical and Clinical Science, University of Exeter, Exeter, UK

Thank you to the authors for responding to and addressing our comments. I have one further comment on the replication analysis using UK Biobank data. The sensitivity analyses which the authors carried out showed that adjusting for covariates prior to inverse-normalization does affect the results. While this does not affect the main conclusions drawn, it may affect the results of the gene-based tests, and in addition, other investigators using the methods as a guide may draw inappropriate conclusions if adjusting for principal components prior to inverse-normalizing their phenotype. Ideally, the UK Biobank analysis should be redone with the appropriate phenotype transformation, and the methods and results sections updated accordingly. However, if the authors consider that such a revision would be too extensive, given that the conclusions do not change, it would at least be helpful to note the issue as a limitation in the discussion and make it clear in the methods that adjusting for covariates (such as principal components) should be done after inverse-normalising the phenotype – so it can be used appropriately by others.

We have read this submission. We believe that we have an appropriate level of expertise to confirm that it is of an acceptable scientific standard.

# Response

**Published:** 2018-07-17

**Authors:** Victoria Jackson

**Affiliations:** University of Leicester, UK

Thank you for you approval of our article, and your additional comment. As suggested, we have added a further limitation to the discussion of the paper outlining the issue regarding the trait transformation. This has also been noted in the methods. We have also included the results of the sensitivity analyses in the supplement (Supplementary Figure 4).

# Peer review: Referee response for version 1

**DOI:** 10.21956/wellcomeopenres.13627.r30984

**Published:** 2018-04-04

**Authors:** Lisa Strug, Naim Panjwani

**Affiliations:** 1 Research Institute, Hospital for Sick Children, Toronto, ON, Canada; 2 The Hospital for Sick Children, Toronto, ON, Canada

The authors have performed a large genome-wide association study in subjects of European (36,998 in the discovery set and 111,556 in the replication set) and African (7,721 in the discovery set) ancestries for various lung function measures: FEV1, FVC and FEV1/FVC ratio. Both common and rare variant analyses are performed, and the effect of smoking on the associations is also assessed. The discovery set consisted of CHARGE and SpiroMeta consortia meta analysis using the Human Exome array, while the replication set consisted of genotypes on the HumanCoreExome array and the UK Biobank’s custom arrays. A total of 7 novel regions were identified by the authors that met the overall (discovery+replication) Bonferroni-adjusted P-value of 2.8x10^-7 after adjustment for various covariates such as age, sex, height, and ancestry using principal components. All identified novel SNPs are of common frequency, and two of the SNPs are in high LD with missense variants predicted to be damaging.

 Some areas for improvement:

  

- Two rare variant tests were chosen and applied to the data as opposed to choosing a combined test (e.g. Derkach et al 2013 Genetic Epidemiology). A combined test would be more powerful.
- The authors should explain why there was an inverse normalization of the traits in SpiroMeta but not in CHARGE, and provide some sensitivity analysis.  
- There appear to be very large differences in Effect Allele Frequencies between the discovery and replication samples. Do the authors have an explanation for this? This might point to local ancestry differences that could be relevant, and should be further investigated.
- The eQTL analysis could formally investigate colocalization as opposed to cross-referencing individual associated SNPs with public repositories, and there are  several different methods that achieve this goal: e.g. COLOC, eCAVIAR, Sherlock, RTC or EnLoc.
- In the replication analyses section, it is stated that “Traits were adjusted for age, age^2, height, sex, ten principal components and pack-years (ever smokers only), and *inverse normally transformed.*” For clarity, the authors should be specific about whether the trait (FEV1, FVC, or FEV1/FVC) was inverse normalized first and age, age^2, sex, 10 PCs were then added as covariates in the genetic association model
- In the methods section for the rare variant testing Skat appears to be incorrectly referred to as a Fisher’s combined method.
- The authors should provide the justification for their various significance criteria used in each of the analyses. 
- The authors should list the MAF alongside the p-values reported in the text for clarity for the single variant analysis results

We have read this submission. We believe that we have an appropriate level of expertise to confirm that it is of an acceptable scientific standard.

# Peer review: Referee response for version 1

**DOI:** 10.21956/wellcomeopenres.13627.r29790

**Published:** 2018-01-25

**Authors:** Rachel M. Freathy, Robin Beaumont

**Affiliations:** 1 Institute of Biomedical and Clinical Science, University of Exeter, Exeter, UK

The authors performed GWAS of FEV1, FVC and FEV1/FVC ratio at 179,215 SNPs from exome arrays. They identified 6 common frequency SNPs associated with at least one of these traits. They also identified 1 SNP in a region with known frequency differences across European populations suggesting that population structure may not have been fully accounted for in their analyses. Strengths of the study include the large sample size and comprehensive approach to assessing associations with low frequency and rare variants. We have the following concerns.

 Main concerns:

1. The phenotypes seem to have been adjusted for covariates and ancestry specific principal components prior to being inverse normally transformed. This transformation has the potential to introduce correlations between principal components and the inverse normally transformed phenotype ( https://www.biorxiv.org/content/early/2017/05/15/137232). Since one of the SNPs identified as being associated with the phenotype is known to vary in frequency across European populations, and the authors note that they cannot rule out the effects of population structure on the identified associations this raises concerns that some of the other associations could also be artefacts driven by failure to properly account for population stratification. It should explicitly be mentioned in the methods whether adjustments were made for ancestry specific principal components prior to inverse normal transforming the phenotype in the SpiroMeta Consortium component of the meta analysis or was included as a covariate in the phenotype - SNP association analysis.
2. Indeed, in the replication analysis in UK Biobank principal components were adjusted for prior to inverse normally transforming the data. Was genotyping chip adjusted for in this cohort (which should be done in the phenotype - SNP analysis)? The UKBiLEVE chip was enriched for smokers, which could affect association analyses unless chip is included as a covariate. In addition the interim data release (which seems to be what is used here - please clarify in the methods whether the data comes from the interim (2015) or full (2017) data release) featured some discrepancies between the two chips, which can introduce spurious associations especially if adjustment is not made for genotyping chip.
3. Why was raw trait used in CHARGE but inverse normalised in SpiroMeta Consortium? This seems an odd choice  

Minor concerns:

1. In the discussion, the authors mention that the 6 identified SNPs not attributed to population structure passed the Bonferroni significance threshold. They then mention that the SNPs ALSO pass Bonferroni corrected significance thresholds in the replication analysis. This could be misleading, since not all SNPs passed the Bonferroni threshold in the discovery only dataset.
2. The authors mention that correction was made for genomic inflation statistic (λ), but we could not find the statistics relating to this. The figures should be given in the manuscript.

We have read this submission. We believe that we have an appropriate level of expertise to confirm that it is of an acceptable scientific standard, however we have significant reservations, as outlined above.
